# Socioeconomic benefit to individuals of achieving 2020 targets for four neglected tropical diseases controlled/eliminated by innovative and intensified disease management: Human African trypanosomiasis, leprosy, visceral leishmaniasis, Chagas disease

**DOI:** 10.1371/journal.pntd.0006250

**Published:** 2018-03-13

**Authors:** Edeltraud J. Lenk, William K. Redekop, Marianne Luyendijk, Christopher Fitzpatrick, Louis Niessen, Wilma A. Stolk, Fabrizio Tediosi, Adriana J. Rijnsburger, Roel Bakker, Jan A. C. Hontelez, Jan H. Richardus, Julie Jacobson, Epke A. Le Rutte, Sake J. de Vlas, Johan L. Severens

**Affiliations:** 1 Erasmus School of Health Policy & Management, Erasmus University Rotterdam, Rotterdam, The Netherlands; 2 Department of control of Neglected Tropical Diseases, World Health Organization, Geneva, Switzerland; 3 Centre for Applied Health Research and Delivery, Department of International Public Health, Liverpool School of Tropical Medicine and University of Liverpool, Liverpool, United Kingdom; 4 Department of Public Health, Erasmus MC, University Medical Center Rotterdam, Rotterdam, The Netherlands; 5 Swiss Tropical and Public Health Institute, University of Basel, Basel, Switzerland; 6 Medical Delta, TU Delft, Delft, The Netherlands; 7 Global Health Program, Bill & Melinda Gates Foundation, Seattle, Washington, United States of America; Texas A&M University College Station, UNITED STATES

## Abstract

**Background:**

The control or elimination of neglected tropical diseases (NTDs) has targets defined by the WHO for 2020, reinforced by the 2012 London Declaration. We estimated the economic impact to individuals of meeting these targets for human African trypanosomiasis, leprosy, visceral leishmaniasis and Chagas disease, NTDs controlled or eliminated by innovative and intensified disease management (IDM).

**Methods:**

A systematic literature review identified information on productivity loss and out-of-pocket payments (OPPs) related to these NTDs, which were combined with projections of the number of people suffering from each NTD, country and year for 2011–2020 and 2021–2030. The ideal scenario in which the WHO’s 2020 targets are met was compared with a counterfactual scenario that assumed the situation of 1990 stayed unaltered. Economic benefit equaled the difference between the two scenarios. Values are reported in 2005 US$, purchasing power parity-adjusted, discounted at 3% per annum from 2010. Probabilistic sensitivity analyses were used to quantify the degree of uncertainty around the base-case impact estimate.

**Results:**

The total global productivity gained for the four IDM-NTDs was I$ 23.1 (I$ 15.9 –I$ 34.0) billion in 2011–2020 and I$ 35.9 (I$ 25.0 –I$ 51.9) billion in 2021–2030 (2.5^th^ and 97.5^th^ percentiles in brackets), corresponding to US$ 10.7 billion (US$ 7.4 –US$ 15.7) and US$ 16.6 billion (US$ 11.6 –US$ 24.0). Reduction in OPPs was I$ 14 billion (US$ 6.7 billion) and I$ 18 billion (US$ 10.4 billion) for the same periods.

**Conclusions:**

We faced important limitations to our work, such as finding no OPPs for leprosy. We had to combine limited data from various sources, heterogeneous background, and of variable quality. Nevertheless, based on conservative assumptions and subsequent uncertainty analyses, we estimate that the benefits of achieving the targets are considerable. Under plausible scenarios, the economic benefits far exceed the necessary investments by endemic country governments and their development partners. Given the higher frequency of NTDs among the poorest households, these investments represent good value for money in the effort to improve well-being, distribute the world’s prosperity more equitably and reduce inequity.

## Author summary

Human African trypanosomiasis, leprosy, visceral leishmaniasis and Chagas disease are neglected tropical diseases (NTDs) controlled or eliminated by innovative and intensified disease management (IDM). We estimated the economic impact of meeting the 2020 targets defined by the WHO for NTD control/elimination, reinforced by the 2012 London Declaration. A systematic literature review identified information on productivity loss and out-of-pocket payments (OPPs) relating to these NTDs. The economic benefit was calculated by combining the estimated disease cases with estimated productivity losses and OPPs resulting from the diseases, from the perspective of affected individuals. Very little information about OPPs and productivity loss due to NTDs was found in the literature. The total global productivity gained by reaching the goals for the four IDM-NTDs was I$ 23.1 billion in the period 2011–2020 and I$ 35.9 billion in the period 2021–2030. The reduction in OPPs was I$ 14 billion and I$ 18 billion for the same periods. In order to estimate the economic impact of meeting the London declaration targets regarding four IDM-NTDs, we had to combine data from various sources, heterogeneous background, and variable quality. Nevertheless, based on conservative assumptions and subsequent uncertainty analyses, we estimate that the benefits of achieving the targets are nearly double the required investments by endemic country governments and their development partners to reach the 2020 targets.

## Introduction

Disadvantaged populations from low- and middle-income countries (LMICs) often have to deal with the health and economic consequences of neglected tropical diseases (NTDs), which can often aggravate their struggles to avoid poverty.[1–12] Chagas disease, human African trypanosomiasis (HAT), leprosy and visceral leishmaniasis (VL) are still difficult and costly to manage and available tools are unsuitable for use in large-scale preventive control programmes. They should be controlled or eliminated by “innovative and intensified disease management” (IDM), as promoted by the World Health Organization (WHO). [13,14] The populations affected by them frequently live in rural or remote areas, thereby limiting access to diagnosis and treatment of both the disease as well as the disabilities they cause.[15]

Efforts of many private and public sector organizations have aimed at increasing the attention, as well as research and funding, given to NTDs. One of the results was the 2012 London Declaration, based on targets set out in the WHO Roadmap for the control and elimination of 10 NTDs by the year 2020.[15–17]

Compared to studies of the epidemiology and health consequences of NTDs, relatively few studies have examined the impact of NTDs on the productivity and out-of-pocket payments (OPPs) of individuals, households, communities and countries.[18,19] There is clear evidence that health improvements positively influence economic welfare and vice-versa. In this sense, apart from addressing the human fundamental right to the highest attainable standard of health, controlling and eliminating NTDs would also have a direct and sustainable effect on the economic growth and financial welfare of the affected populations, and consequently lead to greater national and global prosperity.[9,12,20–22]

Advances in understanding the economic consequences of NTDs could help to further encourage prevention and control actions, assuring funders and policymakers that resources committed to these efforts are a good investment, or at least resulting in increased health policy dialogue.[18,23]

We estimated the economic benefits of reaching the 2020 WHO targets for four IDM diseases: Chagas disease, human African trypanosomiasis, leprosy and visceral leishmaniasis, which meant estimating how much of the economic loss faced by affected individuals due to productivity loss and out-of-pocket payments secondary to these diseases would be avoided by reaching these targets.

## Methods

### General approach and study design

The general approach to estimate the economic benefits is the same as the one used to calculate the benefits of achieving the 2020 WHO targets for NTDs controlled or eliminated by preventive chemotherapy (PCT) described by Redekop et al. [24] This approach follows the concepts used by Chu et al., and a conceptual framework can be seen as Supporting Information 1 (S1 Fig. Conceptual framework). [25]

The Global Burden of Disease Study (GBD) is the most extensive worldwide observational epidemiological study up to now. Mortality and morbidity from major diseases, injuries and risk factors to health are described at the global, national and regional levels. The GBD-2010 data of prevalent cases of the NTDs included in the London Declaration for the years 1990 and 2010 were used as starting points for the calculation of the estimates for other years. They were obtained by interpolating between 1990 and 2010, as presented by de Vlas et al. The prevalent cases until 2030 were estimated by extrapolation under the assumption that the 2020 WHO Roadmap targets would be met and sustained beyond 2020. For each GBD disease sequela, a comparison is made between a counterfactual scenario (which assumed that the epidemiological situation from 1990 regarding NTDs would continue unabated and that the number of cases would increase as a function of overall population growth) and a target achievement scenario (that considers the 2020 targets described in the 2012 London Declaration and described by the WHO being achieved). [26]

We calculated the base case estimates of the benefit for the period between 2011 and 2030 (i.e., the period between ten years before and ten years after the target achievement) instead of the entire period from 1990 to 2030. The economic benefit was calculated by subtracting the costs for the target achievement scenario from the costs of the counterfactual scenario. The economic benefit of each country was combined in order to provide region and global estimates of the economic benefit.

International US$ (constant 2005 dollars) were used to express all estimates in this study. It is a hypothetical unit of currency that has the same purchasing power as the U.S. dollar has in the United States at a given point in time (in this case 2005). It is estimated using purchasing power parity (PPP) exchange rate, defined as the amount of a country’s currency needed to purchase the same amounts of goods and services in the domestic market as one U.S. dollar would buy in the United States. It is a valid measure frequently used to compare estimates between countries. [27,28]

Constant discounting at 3% was applied to both productivity loss and OPPs, using the base year of 2010. Discounting is a mathematical operation to adjust future costs and effects of health-care interventions to the “present value”. When calculating for discounting, for each year (n) in the future the value of costs or benefits is multiplied by (1/(1 + D) ^n^), D being the discount rate. [24,29,30]

Following WHO’s recommendations, the economic benefits from prevented productivity loss and out-of-pocket payments were reported separately. [23] All calculations were performed using Microsoft Excel (version 2010). [31]

### Perspective

Like previous NTD economic impact studies, we used the human capital approach in our study with the perspective of the individual affected by an NTD to analyze the economic costs per GBD sequela, sex and country.

The WHO Guide to Identifying the Economic Consequences of Disease and Injury distinguishes the following cost categories when calculating the microeconomic impact of disease and injury: expenditures on health; labour and productivity losses; effects on human, physical and financial capital formation; non-market impacts such as leisure or caregiver time. We only included the first two in our analyses, incurred by affected individuals during illness in low- and middle-income countries. [23,32–35]

### Countries

All countries referred by the GBD study as endemic for IDM NTDs were included in the analyses [23]. The list of countries per disease can be found using the open-access web-based dissemination tool available at https://erasmusmcmgz.shinyapps.io/dissemination/.

### Literature review

We performed a systematic review of the literature to identify general and country-specific information on productivity loss (indirect labor costs resulting from reduced working hours and economic activity attributable to morbidity) and direct costs incurred by individuals (such as consultation fees, medication, transport, food, assistance, accommodation) due to the 10 NTDs included in the London Declaration. Although in this paper we report on four IDM NTDs, we combined the 10 NTDs in the review, since many papers often refer to several NTDs and describe the economic impact of the related sequelae. Details about the methodology applied to all NTDs and the results regarding the findings on productivity loss related to the NTDs eligible for PC can be found in the review by Lenk et al.[36] In summary, the searched databases included Embase, Medline (OvidSp), Web of Science, Scopus, CINAHL, PubMed publisher, Cochrane, Popline, Lilacs, Scielo and Google Scholar. Websites of relevant organizations (i.e. World Health Organization, the Centre for Neglected Tropical Diseases, the Carter Center) were also screened for relevant grey literature. The search syntax used for each database can be found in the Supporting Information (S1 File. Literature Search Syntax), and the complete list of institutions searched for grey literature can be found in Supporting Information 3 (S2 File. Grey Literature Search). A total of 11,449 articles concerning all 10 NTDs were identified using the database searches. Of these, 5,316 articles remained after duplicates were removed (S1 Table. Results of database searches). We sorted the articles that were related to each particular disease and screened the abstract and title of all papers, examining the full-text version of all articles that provided information on productivity loss or indirect costs. The paucity of studies that provide quantitative estimates of productivity loss and OPPs from NTDs can be seen in Supporting Information 5 (S2 Table. Literature review—results per disease). [36]

### Productivity loss

**Fig 1:**
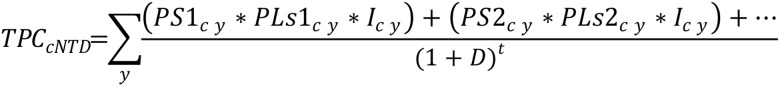
General formula for calculating productivity loss. TPC = Total productivity costs (in US$ 2005), NTD = Neglected Tropical Disease, c = Country, y = Year, PS1 = Number of prevalent cases aged 15+ years with sequela 1, PS2 = Number of prevalent cases aged 15+ years with sequela 2, PLs1 = % productivity loss related to sequela 1 of NTD, PLs2 = % productivity loss related to sequela 2 of NTD, I = GDP per capita in the lowest quintile, D = Annual discount rate, t = Time (years beyond 2010).

The formula below was used to calculate the annual productivity loss for each NTD and country, using the prevalence estimates for the counterfactual and target achievement scenarios independently (Fig 1).

### Prevalent cases

We used the estimates relative to both the counterfactual and the target achievement scenarios calculated by de Vlas et al, as described in the general approach. [26] The population older than 15 years was used for the calculation of productivity loss. [24]

### Productivity loss from disease manifestations

Disease can lead to productivity loss in many ways, including reduced productivity at work (presenteeism), absence from work (absenteeism) or even job loss, which were translated into each infected individual’s annual loss of income due to the effects of each NTD sequela. [23,25]

Unless otherwise specified, we converted the least biased value of productivity loss found in the literature into annual percentages per sequela, assuming 300 working days per year, as seen in Table 1. [25] In every year of the interval we assumed that there were no differences in productivity loss between men and women, between younger and older persons, and between countries. We also assumed that all persons older than 15 years are equally productive. If no estimates for productivity loss were found in the literature, assumptions were made as described in Table 1.

**Table 1:** Annual percentages of productivity loss used in the calculations of economic benefit.

Disease & Sequela	Severity	Base case—Annual productivity loss^1^	Case Mix^2^	Source	Remarks
**Chagas**					
Acute		2.33%	N.A.	[37]	7 of 300 working days
Chronic heart disease		4.67%	N.A.	[37]	14 of 300 working days
Chronic digestive disease	Normal bowel function	0%	30%	[37,38]	1% of the individuals with abnormal bowel function are assumed to undergo surgery, with a productivity loss of 45% (135 days of 100% productivity loss). Weighted average of prod loss of 3.8%.
	Abnormal bowel function	5%	70%	[37,39]	
Heart failure	Mild	0%	10%	[37,40]	14/300 working days and disability weight. Weighted average of prod loss of 61%.
	Moderate	4%	30%		
	Severe	100%	60%		
**Human African trypanosomiasis**					
Cognitive impairment	Severe	100% (Assumption)	52.5%^4^	^7^	Weighted average of productivity loss of 57%.^4^
Disfigurement	Level 2	10%^3^	47.5%^4^	^7^	
**Leprosy**					
Disfigurement due to leprosy	Level 2	28%	N.A.	[41]	
**Visceral leishmaniasis**					
Visceral leishmaniasis		100% (if untreated)^5^	N.A.	[42–45]	Country-specific values were used to reflect differences in diagnosis and/or treatment patterns.
		6–30% (if treated)^6^			

N.A.–Not applicable

^1.^ If the original source did not provide the percentage of productivity loss, this was calculated based on the measurement unit used in the original source.

^2.^ The case mix represents the distribution of the different degrees of severity within a disease sequela. Since the prevalent case estimates were only available per disease sequela and not severity, for sequelae with heterogeneous levels of severity (i.e., mix of milder and more severe forms), the productivity loss values (of the different degrees of severity) were combined with the case mix frequency to calculate a frequency-weighted value of productivity loss for that sequela. For sequelae with a more homogeneous level of severity, the productivity loss value was applied to all prevalent cases.

^3.^ Used the same as onchocerciasis moderate skin disease [46].

^4.^ The case mix in 2010 consisted of 47.5% mild cases and 52.5% severe cases, which changed linearly to 100% mild cases and 0% severe cases in 2020. Consequently, the weighted productivity loss of 57% in 2010 decreased linearly to 10% in 2020, which was represented solely by the productivity loss due to mild cases.”.

^5.^ The global estimate for the productivity loss of untreated patients is 50% (assuming 100% productivity loss over duration of illness, and assumed duration from symptoms to death is 6 months).

^6.^ Productivity loss for treated patients in India, Sudan, Bangladesh, and Nepal is 20%, 30%, 6% and 20% respectively, which was extrapolated to the respective WHO region.

^7.^ Case-mix values from the GBD study documentation and from the assumptions used by de Vlas et al.

Table 1 shows the estimates of annual productivity loss for each sequela included in the GBD study for IDM NTDs. Sequelae related to poor mental illness due to IDM diseases were not included in the GBD study and cutaneous leishmaniasis is not included in the London Declaration. Please refer to S3 Table. Publications reporting productivity loss for Chagas Disease and S4 Table. Publications reporting productivity loss for Visceral Leishmaniasis for a more detailed description of the sources. It shows mostly the productivity loss from absenteeism, due to lack of data on presenteeism. The table also shows additional information for chronic digestive disease and heart failure due to Chagas disease since the GBD study only reported the number of cases per disease sequela and not according to severity level. For example, regarding heart disease, only the numbers of cases with heart disease were reported and not the numbers per severity level (i.e. mild, moderate, severe). Therefore, in order to calculate an overall estimate of productivity loss, we had to combine our estimates of productivity loss per severity level with the estimated frequencies of the different severity levels (i.e., the ‘case mix’). Table 1 therefore shows the productivity loss according to severity and case mix regarding severity for chronic digestive disease and heart failure. Upper and lower limits for the estimates of productivity loss are shown in the sensitivity analysis section.

### Productivity loss due to premature mortality

The number of productive years lost due to NTD-related premature mortality per person was estimated using the country-, age-, and sex-specific data on years-of-life lost (YLL) as provided by the GBD study. The GBD calculations used uniform Japanese life-expectancies attributed to the year of death, but for our study we preferred to use country-specific life expectancies and only for the study period 1990–2030. We have therefore divided the YLL values by the Japanese age-specific life-expectancies to arrive at the number of deaths per country, age and sex, and treated them as incident cases for ‘absent persons’ due to death by an NTD (e.g., visceral leishmaniasis). The prevalence of such ‘absent persons’ was then calculated similar to the procedure for irreversible disease manifestations. Work-years lost were now calculated by the difference between the number of absent persons for the counterfactual scenario and target achievement scenario over the 1990–2030 period. Economic benefit from averted premature mortality in the 2011–2030 period was calculated by combining the lost productive years with income per person, for the 15+ age group. Discounting at 3% was applied to the results using the base year of 2010. [24]

### Income

IDM-NTDs are highly prevalent in countries that are no longer regarded as low-income countries. Nevertheless, most NTDs continue to affect poor populations that do not experience the welfare and health benefits of the economic growth seen in these countries. [47,48]

Different methods were applied in previous economic analyses of NTDs to estimate the rural wage, including use of GDP per capita, average agricultural value added per worker and the lowest wage estimate from distinct predefined wage sources. [37,38,40]

We compared the GDP per capita of the lowest income quintile with the minimum nominal annual wage (both 2010 PPP) for the endemic countries with the highest number of prevalent cases (which would have the highest impact on the final results) and found that the minimum wage was higher than the GDP per capita of the lowest income quintile. Considering the characteristics of the populations affected by NTDs regarding welfare mentioned above, we decided to use the GDP per capita of the lowest income quintile as a proxy for income when calculating the base-case impact estimate, and use only one data source for income instead of several.

GDP per capita for each country (purchase power parity-PPP, 2005 international $) and income shares of the five income quintiles were obtained from the World Development Indicators of the World Bank’s website.[49] In the rare cases where information about GDP per capita or income shares of the year 2010 for a country was lacking, we used data from previous years; if no information from any year was available, we used the average of surrounding countries.

Since many of the countries included in this study have shown an increase in the GDP per capita of the lowest quintile in the last decade, we assumed that the income shares and the GDP per capita remained constant over the assessed period of 2011–2030, to keep estimates conservative. Income was not adjusted for labor force participation (people employed or actively looking for work) or age-related income patterns.

### Out-of-pocket payments (OPPs)

**Fig 2:**
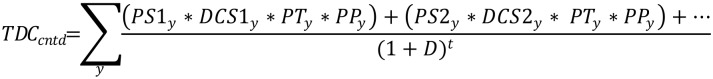
General formula for calculating out-of-pocket payments. TDC = Total out-of-pocket payments (in US$ 2005), NTD = Neglected tropical disease, c = Country, y = Year, PS1 = Number of persons with sequela 1 of NTD, PS2 = Number of persons with sequela 2 of NTD, DCS1 = Annual out-of-pocket payments relating to sequela 1 (per WHO region or country), DCS2 = Annual out-of-pocket payments relating to sequela 2 (per WHO region or country), PT = Percentage of patients treated, PP = Percentage of patients paying for the treatment, D = Annual discount rate, t = Time (years).

The annual economic burden related to out-of-pocket payments was calculated using the formula below for each country and NTD independently (Fig 2). Despite the limited number of studies of OPPs from IDM-NTDs, it was possible to use country-specific values for Brazil, Argentina and Mexico, currently the countries with the highest prevalence of Chagas disease (based on GBD estimates). Their values for OPPs and productivity loss were therefore calculated separately. They serve as examples of how the economic impact of Chagas disease could be calculated for each country, if sufficient country-specific data are available.

Costs were calculated by multiplying four values: the total number of cases in each year; annual direct costs per sequela; percentage of cases treated each year, and percentage of patients paying for treatment each year. Since OPPs are not related to the ability to work, they were calculated for all prevalent cases, including children. We assumed no change in prices for the period 2011–2030 to keep estimates conservative.

### Prevalence estimates

The same prevalence estimates used to calculate the productivity loss were used to calculate the OPPs.

### Annual out-of-pocket payments

OPPs relate to expenses usually incurred by an affected individual due to the illness, including consultation fees, medication, diagnostic tests, travel and escort costs, food, accommodation, etc. Whenever the information was available, the cost of the drug was excluded from the OPPs in case it is donated for free or reimbursed, as well as consultation or laboratory exams if they are also covered by the local health system. Depending on the data identified in the literature, country- or region-specific values were used. The same treatment value per sequela was used for all individuals in each country and sequela, and prices were adjusted to 2005 values using Consumer Price Index (CPI) and purchase power parity (PPP).[49]

In our calculations, the amount paid by patients varied depending on which direct costs patients have to pay per disease sequela and country. OPPs for HAT, for instance, included consultation fees, cost of travel, laboratory costs, all expenses for hospitalization as well as food for the patient and the caregiver. If the OPP described in the literature did not include non-medical payments, since estimates for these costs were lacking in the literature, we opted not to include them, also to keep our results conservative. If the medication was not included in the list of reimbursed drugs or the NTD was not included in the health insurance package, we assumed that all patients had to pay for treatment. This was the case for the three countries for which we could find specific information in the literature about Chagas disease. [38,50–68]

### Percentage of cases treated and paying for treatment

The Sustainable Development Goals (SDGs) emphasize the need to address inequity and provide health for all. The goal of universal health coverage (UHC) means financial risk protection, access to quality healthcare services, and access to safe, effective, quality, and affordable essential medicines and vaccines for all. [69] In line with these concepts, a joint WHO/World Bank framework for monitoring progress towards UHC proposed a target of a minimum of 80% essential health services coverage and 100% financial protection from out-of-pocket payments in 2030, which would mean 100% of the population at risk protected against out-of-pocket payments due to NTDs by 2030. [70]

Parallel to the assumption that the London Declaration targets would be met, we therefore assumed a scenario where 80% of health coverage and 100% of financial protection would be achieved in 2030, instead of only assuming that people seeking care ‘do not suffer financial hardship when using health services.’ [71] We assumed that the percentage of patients currently paying for the treatment corresponds to the percentage of persons not covered by health systems or insurance, since, by definition, out-of-pocket payments are direct payments made by individuals to healthcare providers at the time of service use. [72]

In the counterfactual scenario, the percentage of cases that were treated in 2010 was kept constant at that level until 2030, to simulate a situation where nothing would be done. Similarly, the percentage of cases that paid for their treatment in 2010 was kept constant until 2030. In the target achievement scenario, we assumed that the percentage of cases treated in 2010 would linearly increase to 80% in 2030, and we assumed that the percentage of patients who paid for their treatment in 2010 would linearly decrease to 0% in 2030.

For these percentages, the literature review provided country-specific data for the three most prevalent countries for Chagas disease: Argentina, Brazil and Mexico. [38,53,56,58,60–65,73–75] A general average price for Latin America from Wilson et al was used for all other endemic countries, after correction for PPP for each endemic country (Table 2). [51]

**Table 2:** Out-of-pocket payments, percentage of patients being treated and percentage of patients paying for treatment according to the literature, used in the calculations for Chagas disease (I$—International dollars).

Out-of-pocket payments					
	Acute	Chronic Heart Disease	Chronic Digestive Disease	Heart failure	Source
**Argentina**	$ 32.35	$ 3,505.46	$ 4,275.12	$ 3,505.46	[37,76]
**Brazil**	No costs	$ 2,574.21	$ 902.71	$ 8,231.06	[56,58]
**Mexico**	$ 112.54	$ 267.69	$ 875.90	$ 19,351.39	[68]
**General** ^1^	$ 15.98–46.94	$ 390.1–1115.83	$ 390.1–1115.83	$ 296.2–1564.03	[38,51]
**Percentage being treated**					
**All countries**	10%	35%	35%	35%	[38,39,51]
**Percentage paying for treatment**					
**Argentina**	100%	38%	38%	38%	[54,55,57,77,78]
**Brazil**	0%	25%	25%	25%	[57,79,80]
**Mexico**	100%	100%	100%	100%	[50,57,81,82]
**General** ^2^	100%	25%	25%	25%	[57,79,80]

^1.^ Between country variation

^2.^ For conservative reasons, we assumed the same situation as in Brazil for all other endemic countries, since Brazil has the lowest percentage of people paying: 75% of the population has free access to its health system. [79]

Like Chagas, some country-specific OPPs for visceral leishmaniasis were found for India, Bangladesh, Nepal, and Sudan, which were used to calculate the annual OPPs for these countries (sources listed in S5 Table. Publications reporting Out-of-Pocket Payments for Chagas disease and S6 Table. Publications reporting Out-of-Pocket Payments for Visceral Leishmaniasis). A general average price available from the literature was used for all other endemic countries. Percentages for treated patients (successfully and unsuccessfully), untreated patients and patients paying for treatment were also derived from the literature and were assumed to linearly reach 80% treatment (keeping the same proportion between the three treatment categories) and 100% not paying for treatment in 2030, considering UHC as previously mentioned (Table 3).

**Table 3:** Values used to calculate out-of-pocket payments (OPPs) for visceral leishmaniasis (I$—International dollars).

Out-of-pocket payments		Reference
India	$ 354.75	[43]
Sudan	$ 488.89	[43,83,84]
Bangladesh	$ 286.84	[45]
Nepal	$ 364.00	[42]
General	$ 160.00	[84,85]
**Percentage being treated**		
India	80%	[86–88]
Sudan		
treated successfully	50%	[89]
treated unsuccessfully	5%	
untreated (undetected)	45%	
Nepal/Bangladesh	80%	[87,90]

All drugs currently used for the treatment of human African trypanosomiasis are donated to WHO for free distribution by the manufacturers (Sanofi and Bayer). Nevertheless, individuals affected by HAT still bear other costs than medication costs when seeking treatment, which is one of the reasons for many of them either not to seek treatment, or only do so long after their diagnosis or when their symptoms become more acute. [91] The OPPs described for HAT in the study by Lutumba et al. included these costs, i.e consultation fees, cost of travel, laboratory/diagnostic costs, food for the patient and caregiver during hospitalization, and material such as syringes and needles. These costs were used for all endemic countries, after correction (Consumer Price Index—CPI and purchase power parity—PPP (Table 4). [27,91–93].

**Table 4:** Values used to calculate out-of-pocket payments (OPPs) for human African trypanosomiasis (I$—International dollars).

OPPs		Reference
Annual prices per HAT case	$ 156.77	[91]
**Percentage being treated**		
General	24%	[94] (7,200 reported, 30,000 estimated)
**Patients paying**		
General	100%	Assumption

We assumed no OPPs for leprosy since no information was available from the literature at the time the literature review was performed and to keep the estimates of OPP conservative. This assumption is supported by the fact that multidrug therapy (MDT) has been made available free of charge through the WHO for the past 20 years. The costs of palliative treatment of the incurable sequelae were not included for the same reasons.[70]

### Return on investment

We calculated the net return on investment (ROI) by obtaining a crude estimate of the relationship between the economic benefit and the necessary investments to reach the 2020 NTD Roadmap targets and the 2030 SDG targets. The net ROI is the current value of the benefit to affected individuals minus the current value of the cost to public and philanthropic funders, divided by the current value of the cost to public and philanthropic funders. The economic benefit to affected individuals of averted OPP and productivity loss calculated in this study and the investment costs based on recent WHO estimates published in the Third Report on Neglected Tropical Diseases were used in these calculations.

In the case of IDM NTDs, only investments in individual management of HAT, leprosy, and visceral leishmaniasis were included, as well as active case finding for HAT, leprosy and VL, and vector control for VL (only in areas of the Indian subcontinent that are not co-endemic with malaria), plus the cost of integrated surveillance in HAT-endemic areas. Investments and benefits related to Chagas disease were not included. [70]

For comparison to the disease-specific investment targets published by WHO in the Third Report on Neglected Tropical Diseases, the I$ 2010 benefits were converted to US$ 2015. Since part of these benefits can clearly be credited to investments made before 2011, we conservatively assumed the investments to be equal to those in 2011 (adjusted for inflation). We assumed 1990 to mark the beginning of concerted global efforts to control most NTDs and 2011 to mark the beginning of the recent scale-up in investment to eliminate them. In reality, investments before 2011 were probably lower than this in most countries. We did not consider investments in improving housing and water and sanitation that occurred over the same period, since these were not targeted at the NTDs but contributed to their control nonetheless. The ROI for middle and low income settings was not calculated separately due to lack of the necessary data on investments. Since investments estimates are given in US$, ROI is presented in US$ only. A discount rate of 3% per annum was applied for both costs and benefits. More detailed information on the ROI and the internal rate of return per WHO region, as well as other considerations on the investment case of ending/controlling NTD, can be found in the recently published DCP3/World Bank volume on infectious diseases by Fitzpatrick et al. [95]

### Sensitivity analysis

The economic benefit was calculated using base-case values for the components of the formulae described above. We examined how much effect changes in four input parameters used in our calculations had on the estimated economic benefit: 1) the prevalence estimates, 2) the productivity loss percentages and out-of-pocket payments, 3) income, and 4) percentage of patients seeking and paying for treatment.

We performed a probabilistic sensitivity analysis, where the values of all input parameters are varied simultaneously to obtain the overall uncertainty regarding the economic benefit. Beta PERT distributions were used in combination with values shown in Table 5; the values of the different parameters were assumed to be independent of each other.

**Table 5:** Upper and lower limits used in the sensitivity analyses.

	Chagas disease			HAT			Leprosy			Visceral leishmaniasis		
	Lower limit	Point estimate	Upper limit	Lower limit	Point estimate	Upper limit	Lower limit	Point estimate	Upper limit	Lower limit	Point estimate	Upper limit
**Relative uncertainty in global prevalence in 2010**	0.226	1.000	1.90	0.190	1.000	2.90	0.689	1.000	1.41	0.569	1.000	1.57
**Estimates of productivity loss** ^1^	50%	57%	100%	2%	5%	10%	14%	28%	55%	6%	19%	100%
**Estimates of income**	0.836	1.000	1.673	0.588	1.000	2.265	0.871	1.000	1.424	0.871	1.000	1.424
**Out-of-Pocket Payments per person**	0.50	1.00	2.00	115	700	11,954	N.A.^6^	N.A. ^6^	N.A. ^6^	1.00	1.00	1.00
**Probability of being treated (counterfactual scenario)**	0%	6.7% ^2^ 35% ^3^^,^^4^^,^^5^	100%	0%	24%	100%	N.A. ^6^	N.A. ^6^	N.A. ^6^	0%	55%	100%
**Probability of paying for healthcare** **(counterfactual scenario)**	0%	67% ^2^ 69.4% ^3^^,^^4^ 34.2% ^5^	100%	0%	80%	100%	N.A. ^6^	N.A. ^6^	N.A. ^6^	0%	80%	100%
**Probability of being treated** **(target achievement scenario)**	0%	80 ^2^ ^3^^,^^4^^,^^5^	100%	0%	80%	100%	N.A. ^6^	N.A. ^6^	N.A. ^6^	0%	80%	100%
**Probability of paying for healthcare** **(target achievement scenario)**	0%	0%	100%	0%	0%	100%	N.A. ^6^	N.A. ^6^	N.A. ^6^	0%	0%	100%

^1.^ The productivity loss estimates seen in Table 1 are here shown as frequency-weighted estimates per disease with their respective upper and lower limits used in the sensitivity analysis

^2.^ Value for acute Chagas disease sequela (weighted average of three most prevalent countries).

^3.^ Value for chronic heart disease sequela (weighted average of three most prevalent countries).

^4.^ Value for chronic digestive disease sequela (weighted average of three most prevalent countries).

^5.^ Value for heart failure sequela (weighted average of three most prevalent countries).

^6.^ N.A.–not applicable

By applying the country-specific upper and lower confidence limits of the GBD—2010 estimates, we examined the relevance of uncertainty about the prevalence in all the years in the 2010–2030 period. Productivity loss and OPP values were varied by using the highest and lowest values found in articles with sufficient quality retrieved in the literature review. If no estimates were available from the literature, assumptions were made, as described in Table 1. For each disease, we varied income using data from the country with the most prevalent cases in the world. The lower limit of income equalled the average income in the lowest income decile in that country, while the upper limit equalled the average income in the second-lowest income quintile. For OPPs, we varied the uncertainty regarding out-of-pocket payments (per person) by a factor of 2 (i.e., from 50% to 200%). [24]

The rough estimates of the return on investment calculated in this study were not subject to sensitivity analysis, following the original publication by Fitzpatrick et al. [95].

### Technical validity

R scripts were written to examine the technical validity of our Excel-based calculations. They used the same original data (GBD, UNPOP (United Nations Population Division), GDP, productivity loss) as the Excel files (though transformed), but were completely independent of the Excel calculations. The small number of differences were found led to the improvement of the formulae for some of the diseases and subsequent matching (or calibration) of the results, although the general programming in Excel did not change. R scripts and sample Excel sheets can be found in the Supporting Information section.

The health benefits calculated by De Vlas et al. and the economic benefits shown here will be publicly available through the open access website: https://erasmusmcmgz.shinyapps.io/dissemination/.

## Results

### Productivity loss

**Fig 3:**
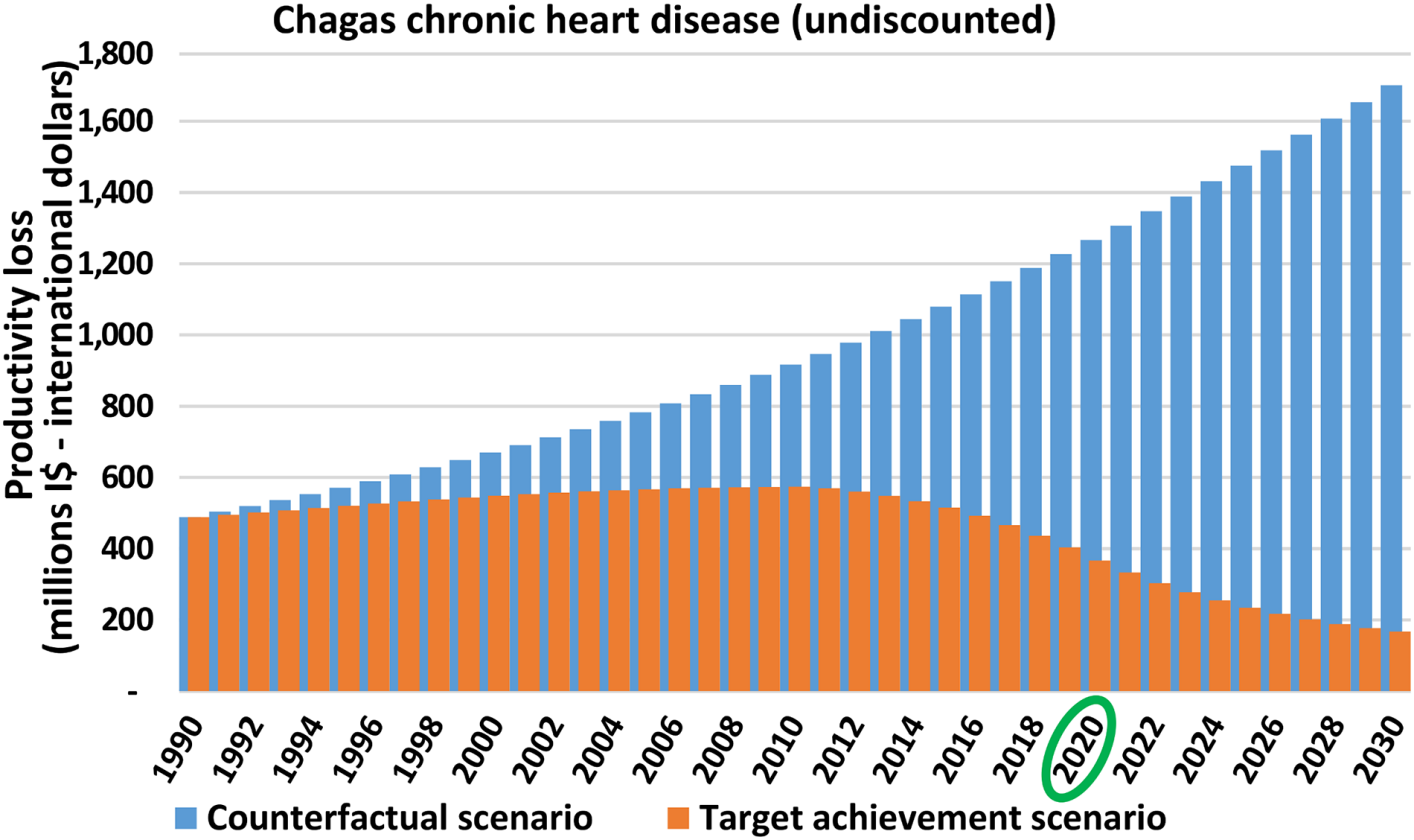
Productivity loss due to Chagas chronic heart disease according to the counterfactual and target achievement scenarios (millions I$—International dollars). Total global loss per year in the counterfactual scenario (blue) and target achievement scenario (orange). The economic benefit is the difference between both scenarios.

Fig 3 provides a graphical demonstration of the different cost estimates and their trend over time in the counterfactual and target achievement scenarios. The difference between the rising productivity costs in the counterfactual scenario and the decreasing costs in the target achievement scenario represents the total economic benefit of achieving the targets, which is highly dependent on the estimated prevalence of the IDM-NTDs over time. Since the same pattern can be seen for all IDM diseases and related sequelae, we provide the example of productivity costs from the Chagas chronic heart disease sequela (the sequela with the biggest impact).

### Overview of the global estimates of the economic benefit from averted productivity loss

Table 6 shows the total economic benefits in productivity gain for each of the IDM-NTDs and their sequelae. The total benefits of achieving the targets for all four IDM diseases were estimated at I$ 23.1 billion (I$ 15.9–34.0 billion) or US$ 10.7 billion (US$ 7.4–15.7 billion) in 2011–2020 and I$ 35.9 billion (I$ 25.0–51.9 billion) or US$ 16.6 billion (US$ 11.6–24.0 billion) dollars in 2021–2030 (base case estimates and 2.5^th^ and 97.5^th^ percentile values from the sensitivity analysis).

**Table 6:** Total economic benefit from productivity loss averted, base case estimates and 2.5^th^ and 97.5^th^ percentiles (billions I$—International dollars and US$—US dollars 3% discounting from 2010).

Disease	Sequelae	Economic benefit (productivity loss averted) I$—International dollars		Economic benefit (productivity loss averted) US$—US dollars	
		2011–2020	2021–2030	2011–2020	2021–2030
**Chagas disease**	Acute	$ 0.4	$ 0.5	$ 0.2	$ 0.3
	Chronic heart disease	$ 5.1	$ 7.9	$ 2.9	$ 4.6
	Chronic digestive disease	$ 0.8	$ 1.1	$ 0.5	$ 0.6
	Heart failure	$ 0.3	$ 0.8	$ 0.2	$ 0.5
	Chagas deaths	$ 1.6	$ 2.7	$ 0.9	$ 1.5
	**Total**	$ 8.2 [3.0–17.2]	$ 13.0 [4.9–27.6]	$ 4.7 [1.7–9.8]	$ 7.5 [2.83–15.9]
**HAT**	African trypanosomiasis	$ 0.5	$ 0.6	$ 0.3	$ 0.3
	African trypanosomiasis deaths	$ 2.7	$ 4.1	$ 1.5	$ 2.3
	**Total**	$ 3.2 [2.6–16.6]	$ 4.7 [1.5–9.8]	$ 1.8 [1.5–9.3]	$ 2.6 [0.9–5.5]
**Leprosy**	Disfigurement	$ 3.7	$ 5.0	$ 1.5	$ 2.0
	**Total**	$ 3.7 [2.0–6.2]	$ 5.0 [2.7–8.4]	$ 1.5 [0.8–2.5]	$ 2.0 [1.1–3.4]
**Visceral leishmaniasis**	Visceral leishmaniasis	$ 0.1	$ 0.1	$ 0.03	$ 0.04
	Visceral leishmaniasis deaths	$ 7.9	$ 13.2	$ 2.7	$ 4.5
	**Total**	$ 8.0 [5.1–11.7]	$ 13.3 [8.5–19.4]	$ 2.7 [1.7–3.9]	$ 4.5 [2.9–6.6]
**Total (all diseases)**		**$ 23.1 [15.9–34.0]**	**$ 35.9 [25.0–51.9]**	**$ 10.7 [7.4–15.7]**	**$ 16.6 [11.6–24.0]**

**Fig 4:**
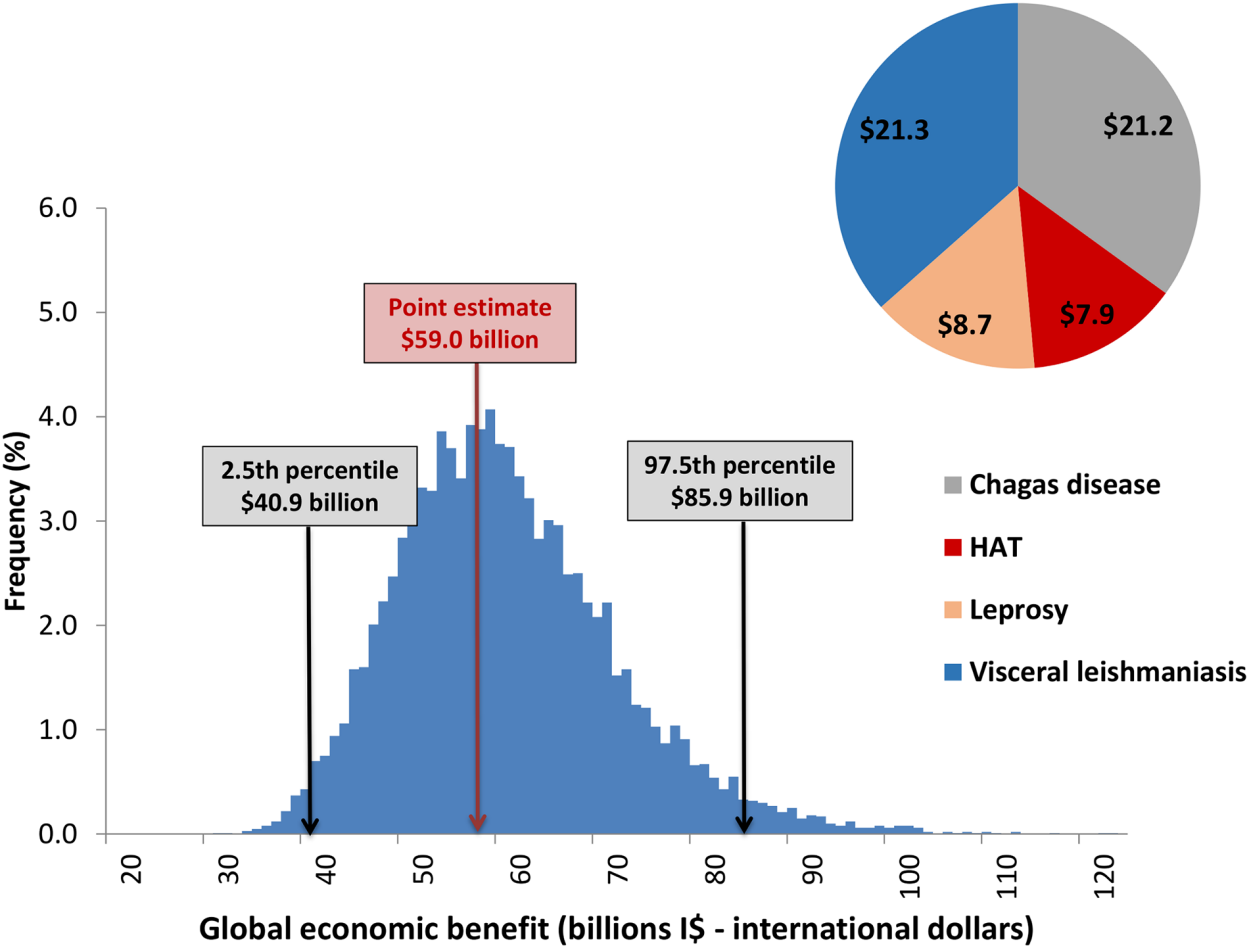
Global economic benefit (productivity loss averted) for IDM NTDs, for the period 2011–2030 (billions I$—International dollars). Global economic benefit from reaching the targets for IDM NTDs, lower and upper estimates from sensitivity analysis. Global economic benefit per disease.

Fig 4 shows the total values per disease (in I$) together with the sensitivity analysis diagram of the calculations of the total economic benefit of achieving the 2020 targets for the IDM diseases. The total economic benefit calculated for the entire period was $59.0 billion, with the 2.5th and 97.5th percentile values of I$ 40.9 and I$ 85.9 billion calculated in the sensitivity analysis.

**Fig 5:**
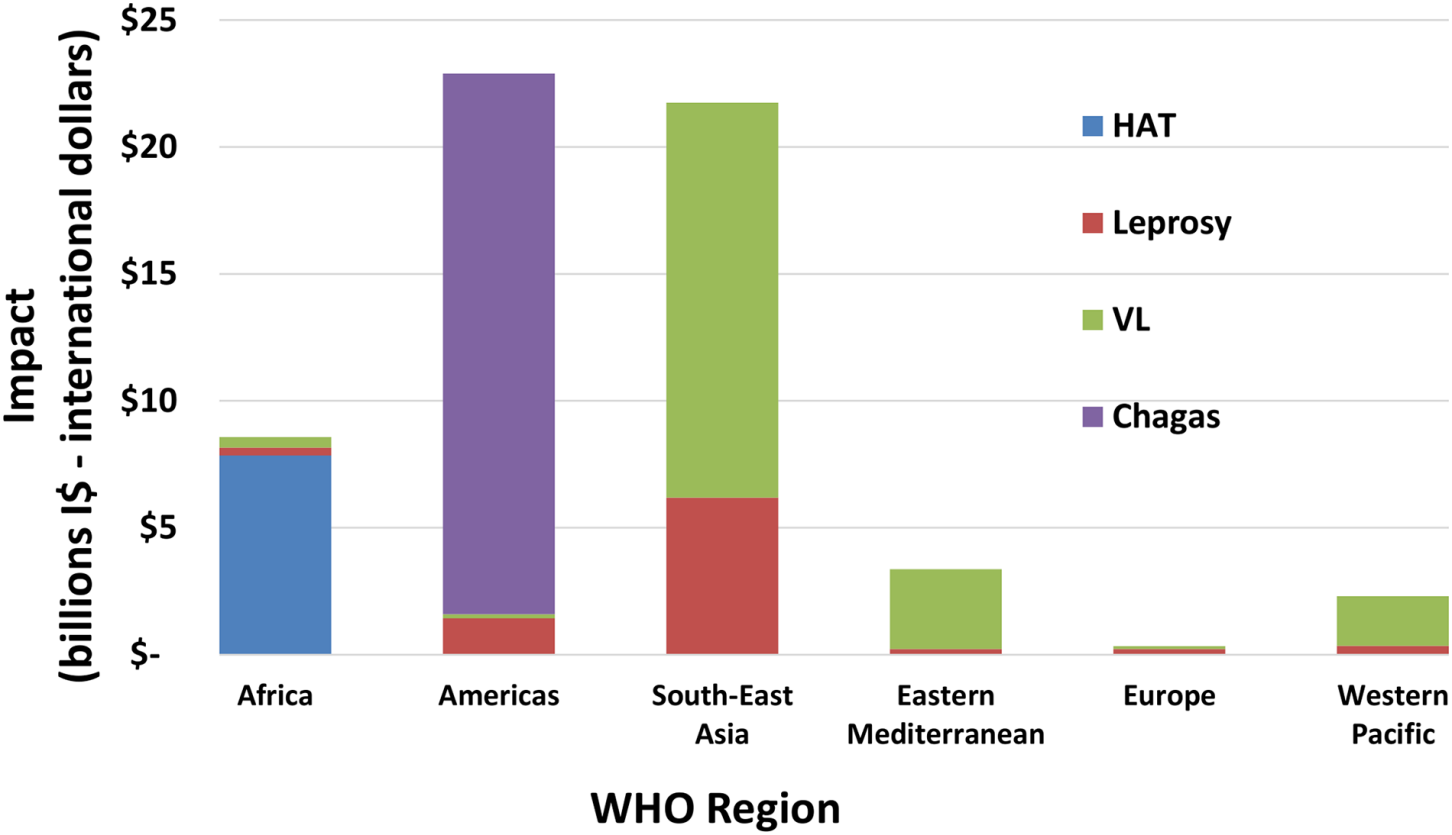
Regional economic benefit (productivity loss averted) for IDM NTDs, for the period 2011–2030 (billions I$—International dollars) per WHO region. Regional economic benefit from reaching the targets for IDM NTDs, for the period 2011–2030 per WHO region.

Fig 5 shows the regional variation in the economic benefit, with the Americas and South-East Asia outweighing over the other regions due to Chagas disease and visceral leishmaniasis, respectively. More productivity loss prevented can be expected in the Americas and South-East Asia regions due to Chagas disease and visceral leishmaniasis, respectively.

### Out-of-pocket payments

IDM-NTDs impose a considerable burden on patients, mostly due to their incurable sequelae, and often compel patients to seek and pay for treatment. [13,96] Table 7 shows the economic gains regarding out-of-pocket payments that could be expected by reaching the 2020 targets for IDM-NTDs. Chagas chronic heart disease is the main reason for the OPPs among all sequelae.

**Table 7:** Total economic benefit from out-of-pocket payments averted, base case estimates and 2.5^th^ and 97.5^th^ percentiles (billions I$—International dollars and US$—US dollars) discounting 3% from 2010.

Disease	Sequelae	Economic benefit (OPPs averted) I$—International dollars		Economic benefit (OPPs averted) US$—US dollars	
		2011–2020	2021–2030	2011–2020	2021–2030
**Chagas disease**	Acute	$ 0.02	$ 0.05	$ 0.01	$ 0.03
	Chronic heart disease	$ 12.52	$ 14.50	$ 5.70	$ 8.20
	Chronic digestive disease	$ 1.41	$ 2.95	$ 0.81	$ 1.74
	Heart failure	$ 0.15	$ 0.48	$ 0.08	$ 0.26
	Total	$ 14.10 [2.2–41.7]	$ 17.97 [2.5–48.6]	$ 6.57 [1.2–21.9]	$ 10.24 [1.3–25.5]
**HAT**	African trypanosomiasis	$ 0.19 [0.001–1.5]	$ 0.20 [0.001–1.6]	$ 0.10 [0.0005–0.75]	$ 0.10 [0.0005–0.80]
**Visceral leishmaniasis**	Visceral leishmaniasis	$ 0.13 [0.06–0.19]	$ 0.14 [0.06–0.22]	$ 0.05 [0.02–0.07]	$ 0.05 [0.02–0.08]
**Total (all diseases)**		$ 14.42 [2.4–42.0]	$ 18.31 [2.6–48.9]	$ 6.72 [1.12–19.55]	$ 10.39 [1.48–27.75]

**Fig 6:**
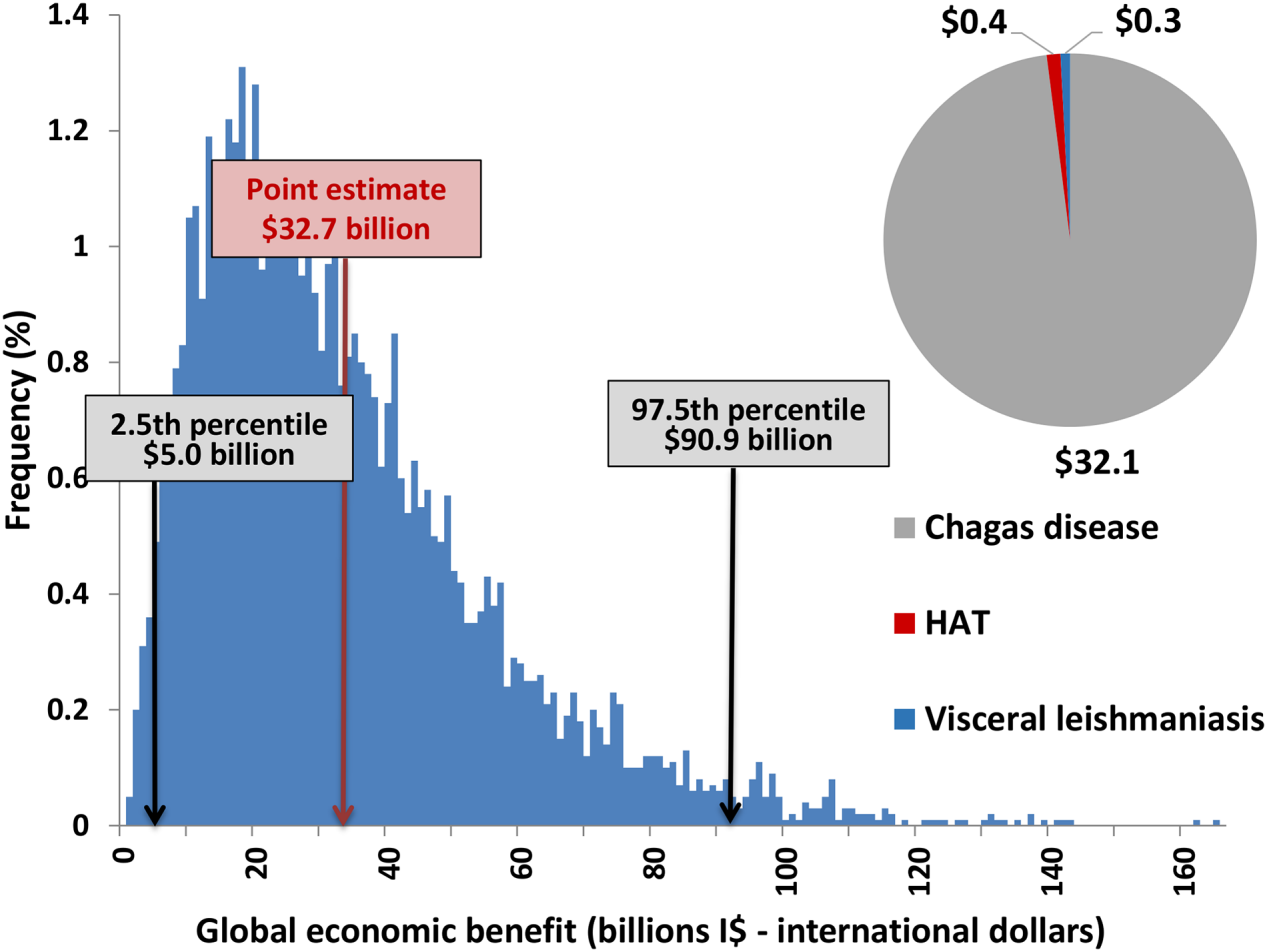
Global economic benefit (out-of-pocket payments averted) for IDM NTDs, for the period 2011–2030 (billions I$—International dollars). Total economic benefit from out-of-pocket payments averted, base case estimates and 2.5th and 97.5th percentiles (billions I$—international dollars), discounting 3% from 2010.

Fig 6 shows the total values per disease together with the sensitivity analysis diagram. The total economic benefit calculated for the entire period was I$ 33 billion, with the 2.5th and 97.5^th^ percentile values of I$ 5 and I$ 90 billion calculated in the sensitivity analysis.

### Return on investment

The ROI was calculated based on an estimated benefit of US$ 5.4 billion (in 2015–2020) and US$ 20.9 billion (in 2015–2030), assuming the 2020 targets for IDM diseases were to be met, and considering costs to funders of US$ 1.1 billion and US$ 2.2 billion in 2015–2020 and 2015–2030, respectively.

The net benefit was estimated to be US$ 0.9 [0.62–1.32] for every dollar invested during the period 1990–2020 and US$ 2.8 [1.94–4.05] for every dollar invested in the period 1990–2030 (best estimates and 2.5^th^ and 97.5^th^ percentiles related only to the benefits). A net benefit of US$ 0.9 per dollar means every dollar invested yielded US$ 1.9 in benefits, or benefits nearly double the investment in the period 1990–2020. [95]

## Discussion

This study is a first attempt to estimate the global economic benefits of achieving the London declaration targets for four IDM NTDs (Chagas disease, human African trypanosomiasis, leprosy, and visceral leishmaniasis).

### General approach

Scarce and heterogeneous data available on country- or regional-specific productivity loss and OPPs related to the different NTDs and their sequelae limits the comprehensiveness of this economic analysis to some extent. The same can be said about the paucity of information regarding the characteristics of the affected populations (e.g. income) and the impact of assumptions regarding the future frequencies of each NTD. More accurate measures of productivity loss caused by NTDs and better descriptions of the affected populations (e.g., type of work, income) in the different affected countries would greatly improve the quality of any estimates of the economic burden of NTDs and the economic benefits of controlling NTDs.

The economic benefits of reaching the 2020 targets for IDM-NTDs were estimated using the human capital approach, which increases comparability with other studies. However, critics may argue that it overestimates the extent of productivity loss from the societal perspective. [97] Nevertheless, the perspective from the individuals affected by these diseases, rather than a societal one, was chosen for this study. The first reason for this choice was to maintain comparability with the recently published results regarding the economic benefit of reaching the 2020 London Declaration targets for PCT NTDs (i.e., the ones controlled or eliminated through preventive chemotherapy).[24] Secondly, the friction cost method focuses only on lost productivity until a replacement can be found. Use of the friction cost method is likely to have a limited effect on the results for leprosy and Chagas since these diseases can lead to reduced productivity while working (presenteeism) rather than simply lost work days (absenteeism) and presenteeism will not necessarily lead to replacement of the worker. Therefore, the use of the friction method is not expected to have a substantial effect on the estimated economic benefit for these diseases. In contrast, the biggest economic impact for HAT and VL is through avoiding premature deaths. Therefore, the friction cost method might have been a better choice, since premature death would lead to worker replacement. We also acknowledge that the aggregation of individual costs does not exactly correspond to the total societal cost, since individuals’ productivity losses can be compensated by a variety of mechanisms at the societal level. Some of these mechanisms are discussed later in this section (e.g., household coping strategies).

Labor is not the only factor that influences the link between income and productivity. In all retrieved papers there was not enough information on whether there is a linear relationship between health and productivity. Therefore, and also because of using conservative values for productivity loss, we assumed that average productivity gain would equal marginal productivity gain. The lack of information in the literature prevents us from knowing if this would have under- or overestimated the results.

Household coping strategies, social security, productivity loss of caregivers of people with IDM-NTDs, and work compensation mechanisms were not included in the calculations. However, household coping strategies can mediate the effects that an illness of a family member can have on household finances in several ways; for example, another member might start working to reduce the loss of household income. However, even if coping strategies are able to maintain the household income, they may reduce future opportunities for children who suspend their education and start working. [23] The productivity loss due to psychosocial consequences of the diseases (i.e.stigma and discrimination) was also not included, since these types of sequelae are not included in the GBD study; their omission may have led to an underestimation of the economic benefit.

The basis for the diseases (and their sequelae) included in the GBD study was a set of brief lay descriptions emphasizing the main functional consequences and symptoms associated with each health state. For the sake of simplicity, comprehensibility, and feasibility, some aspects of health states were inevitably omitted in the GBD study, which means that it might not encompass all disease consequences. [40] For instance, erythema nodosum leprosum, a complication of leprosy that is known to result in direct costs by affected individuals, was not included. [98] Inclusion of other health states would lead to a higher estimate of the productivity loss and OPPs, and consequently, of the economic benefit.

The large differences in the magnitude of the economic gain between the different diseases and sequelae and the affected countries/regions are a direct consequence of the prevalence of each disease in each country, the consequences of each sequela, the chosen proxy for income and the percentages of patients being treated and paying for treatment for these diseases in each country or region.[34] One should be careful when making comparisons between diseases. First, the results for each disease are affected differently by the data limitations and potential biases in the methodology mentioned in the ‘Limitations’ section. The estimated economic benefit from investments in NTDs also relies on the way productivity is valued in monetary terms. Assigning exclusively monetary value to these domains in terms of productivity gain undervalues the much bigger gain in physical and mental health that will lead to the increase in productivity. Therefore, policymaking should consider the health impact of controlling NTDs and not simply the economic benefit (as calculated here).

The expected benefits in 2021–2030 are greater than that in 2011–2020, which is not surprising, since the difference in disease frequency between the counterfactual scenario and target achievement scenario will be greater in 2021–2030 (after the targets are met).

The gains of achieving the 2020 targets for IDM diseases are not restricted to the economic benefits, with billions of people gaining physical and mental health, increased mobility, improved performance in school, access to care, structural improvement of health care services, community participation, and democracy with the control/elimination and eradication of NTDs. [99] Therefore the economic benefits estimated in this study represent just one part of the benefits that society could experience by achieving the London Declaration goals.

### Comparisons with the literature

As far as we are aware of, this is the first reported attempt to estimate the economic burden of IDM diseases per endemic country and globally, from an individual’s perspective. We know of only one other global cost-of-illness study of an IDM disease. Specifically, Lee at al. estimated a global annual burden from Chagas disease of US$ 0.5 ($0.2–1) billion in healthcare costs (corrected to US$2005 values for comparison) and US$ 6 (4–8) billion including lost productivity for Latin America related to disease-induced early mortality, using a societal perspective. Their estimate of healthcare costs is approximately 30% less than our estimate of annual OPPs of US$ 0.85 (0.1–2.4) billion (2011–2030) while their estimate of lost productivity related to mortality is two times greater than our annual general productivity loss average of US$ 1.4 (0.9–2.0) billion using an individual’s perspective. However, it is difficult to make meaningful comparisons between our estimates and their estimates because of differences in methodology. For example, Lee et al grouped countries into quartiles based on GDP per capita (low/low-middle/high-middle/high income) and estimated the healthcare costs by quartile, assuming a linear correlation between costs and GDP per capita. With the exception of the three most prevalent countries (for which we had country-specific estimates), we converted one general Latin American estimate to local currencies of each of the other endemic countries. Lee et al included treatment-seeking probability in their model that was much higher than the percentage that we used (78% versus 35%). Also, they did not mention what they used regarding percentage of people paying for the treatment. [100]

### NTDs and poverty

Paying for treatment—especially for IDM-NTDs treatment—can be catastrophic for individuals and even for households.[35] This is one of the reasons why the Sustainable Development Goals (SDGs) are more difficult to achieve without addressing NTDs (and vice-versa). This is especially true for goal 3.3: ‘By 2030, end the epidemics of AIDS, tuberculosis, malaria and neglected tropical diseases and combat hepatitis, water-borne diseases and other communicable diseases.’ [7,101] Furthermore, NTDs constitute an obstacle to achieving wider human development outcomes (for instance, food and nutritional security, and improved maternal and child health). So, undoubtedly, achieving universal health coverage for NTDs will support progress in various interdependent development areas, such as poverty, education, sanitation, nutrition, water and gender equality. [9,70]

### Limitations

#### Prevalent cases

Due to the scarcity of data on NTD spread and control, the prevalence estimates from the GBD study show very wide confidence intervals. These wide CIs affect the predictions by De Vlas et al. of the health impact of achieving the targets described in the London Declaration. Substantial uncertainty regarding the frequency of IDM diseases also existed, where the variation ranged from five times less to up to three times greater than the mean values (Table 7), dependent on availability of country and disease-specific epidemiological data [26]. This uncertainty only increased as the GBD numbers were extrapolated to estimate the prevalence in the period of 2010–2030. Given the influence of disease frequency on our estimates of economic impact, we included uncertainty about disease frequency in our sensitivity analyses. The results of these analyses show that even if the true prevalence values were close to the lower limits of the ranges used in our analyses, the economic impact would still be substantial.

#### Productivity loss and out-of-pocket payments

In general, limited data was found on productivity loss and out-of-pocket payments, as well as the percentages of patients getting treatment or having to pay for it. As described in the Methods section, the OPP estimates available in the literature regarding IDM-NTDs varied, partly because some sources included only drugs, while others included other medical and non-medical costs. This means that the values used might have been overestimated for some diseases but underestimated for other diseases. For instance, the variability in OPP prices for Chagas disease between Argentina and Brazil is due to their differences regarding the organization of the health system, the approaches to treating Chagas disease and its manifestations, and the infrastructure to treat Chagas manifestations. As an example, the digestive form of Chagas disease frequently needs surgery, but the direct costs related to it depend on access to hospital care and whether treatment is insured. [38,50–68]

We also assumed that the percentage of patients paying for treatment equals the percentage of uninsured persons. This could lead to an overestimation of the costs, since not all individuals affected by NTDs might want treatment or be able to pay for it. However, it could also be an underestimation of the costs, since in many countries insured patients pay for treatment out-of-pocket in order to be treated faster.

When no OPP or productivity loss values could be found, assumptions were made. In some cases, data from other diseases with similar sequelae were used. For instance, the productivity loss from disfigurement due to HAT was estimated using the estimate for onchocerciasis moderate scriot disease since they have the same GBD sequela category. [102]

Most of the studies described lost productivity in working days missed because of the disease sequelae (absenteeism), especially for visceral leishmaniasis and Chagas digestive disease, where treatment itself requires a long hospitalization.[41–43,88,103–106] Only a few studies provided quantitative estimates of the decreased productivity loss at work due to presenteeism, something that can occur because of problems such as disfigurement or the early stages of Chagas disease.[38,62]

Several assumptions regarding the generalizability of data had to be made due to lack of data. First, the same estimate for productivity loss was applied to men and women, even though the degree of productivity loss may differ between the two. [107–112] Secondly, for each sequela, the same estimate for productivity loss was applied to all individuals and countries, even though it differs between professions and settings. A similar situation was seen with OPPs, since sometimes only one estimate for one country was available (i.e. HAT), although the values used in the different countries were adjusted using CPI and PPP. Transferring the data from one country to another might have led to over- or underestimates of productivity loss estimates, as well as OPPs, depending on the disease sequela, the profession and the working environment, and the characteristics of each health care system.[34,113]

The achievement of 80% essential health services coverage and 100% financial protection from out-of-pocket payments in 2030, will surely happen in various ways and paces in the 191 countries included in this study. Most countries might want to invest sooner to gain more efficiency by reducing the vectors and prevalent cases and thereby reducing the number of infected individuals and disease transmission. This approach would probably follow a curve of ‘economies of scale’, which would mean more investments than the linear approach we followed. This would probably result in less economic benefit, but it would be difficult to estimate how much less, given the many possibilities and scenarios each country faces. In this sense, we calculated an ideal scenario that included reaching both the London Declaration targets and the WHO/World Bank recommendations for universal coverage and financial protection regarding NTDs aiming to provide extra arguments in favor of pursuing them.

#### Income

Different methods have been used in previous economic analyses of NTDs to approximate the rural adult wage, including use of GDP per capita, average agricultural value added per worker and the lowest wage rate from different predefined wage sources.[25,114,115]

The GDP per capita of the lowest income quintile was used as a proxy for income for our calculations, since it provided the lowest—and therefore most conservative—estimates possible without having to combine multiple data sources. This approach could be criticized as an overestimation of the annual productivity loss of the affected populations, since NTDs are typically known as diseases of the poorest. We consequently varied income to the GDP per capita of the lowest decile in the sensitivity analysis.

### Sensitivity analyses

A good estimate of the uncertainty around the economic impact shown in this paper is an impossible task, since the uncertainty regarding the impact on prevalence over the years cannot be reliably estimated Although sensitivity analyses were carried out to estimate the degree of uncertainty surrounding our estimates of economic benefit, greater knowledge of the variables used would improve the quality of the analyses.

Despite the effort to account for many of the differences between diseases (sequela, productivity loss, OPP per disease, etc), our analyses represent a simplification of reality. In case more empirical data become available, more detailed studies could be done for each disease. Moreover, individual countries are encouraged to perform similar analyses to gather and use local data wherever possible to derive better local estimates of economic benefit.

Reaching the 2020 targets for the 10 London Declaration NTDs described by the WHO [15,17] will depend on continued and sufficient efforts to achieve them, so especulating that they will be met of course implies a natural uncertainty about the future. The real economic benefit will certainly depend on local data, local circumstances, and the degree that each country will reach those targets. In this sense, our results support the implementation of efforts to reach these goals, but our results cannot help in deciding on how to reach them.

### Conclusions

While the different factors of uncertainty described in the Discussion suggest that the results of this study should be interpreted with care, we can safely conclude that the economic benefits to individuals are at least equal to the investments required by governments and their development partners to reach the London Declaration 2020 targets. It is more likely that the economic benefits will far exceed the necessary investments. Given the higher frequency of NTDs among the poorest households, these investments represent good value for money in efforts to increase human well-being and freedom, to better share the world’s prosperity and reduce inequity. We hope that these results can help policymakers in affected countries to choose to add NTDs to their public health, medical and scientific priority lists. A concerted effort is needed to collect better epidemiological and economic data to enable more accurate and complete estimates, which can be a better basis for planning and decision making.

## Supporting information

S1 Fig Conceptual framework. (PDF)

S1 File Literature search syntax. (PDF)

S2 File Grey literature search. (PDF)

S3 File Excel example of calculations of the counterfactual scenario for onchocerciasis skin disease. (XLSM)

S4 File Excel example of calculations of the remaining case scenario for onchocerciasis skin disease. (XLSM)

S5 File R scripts for the calculations of the counterfactual and remaining case scenarios for onchocerciasis. (ZIP)

S1 Table Results of database searches. The literature review yielded 11449 hits, 5316 after duplicates were excluded, for the 10 NTDs cited in the London Declaration.
(PDF)

S2 Table Literature review—Results per disease. The paucity of studies that provide quantitative estimates of productivity loss and OPPs from NTDs can be seen in the table above.
(PDF)

S3 Table Publications reporting productivity loss for Chagas disease. NA—Not applicable
NR—Not reported
PAHO—Panamerican Health Organization.
(PDF)

S4 Table Publications reporting productivity loss for visceral leishmaniasis. CBA—cost-benefit analysis; CBA—cost-effectiveness analysis; n/a—not applicable; HH = household
1) 3 hospitals initially selected but 1 did not have any patients at time of treatment
2) PKDL Post–kala-azar dermal leishmaniasis, is a complication of visceral Leishmaniasis characterized by a macular, papular, or nodular rash, PKDL develops months to years after apparently successful treatment of kala-azar or in rare cases, in the absence of clinical visceral leishmaniasis.
3) definition of past and current patients: past PKDL case patient was defined as an individual with a macular, papular, or nodular rash for at least 1 month that was diagnosed by a physician and treated with SAG with resolution. A current PKDL case patient was defined as an individual with a macular, popular, or nodular rash for at least 1 month that was diagnosed by the study physician who was experienced in the clinical diagnosis of the disease.
4) excluded: pt kala-azar treatment failure or post-kala-azar-dermal leishmaniasis.
(PDF)

S5 Table Publications reporting out-of-pocket payments for Chagas disease. N.A.–Not applicable
N.R.–Not reported.
(PDF)

S6 Table Publications reporting out-of-pocket payments for visceral leishmaniasis. N.A.–Not applicable
VL—Visceral Leishmaniasis
PKDL—Post–kala-azar dermal leishmaniasis, a complication of visceral Leishmaniasis characterized by a macular, papular, or nodular rash, PKDL develops months to years after apparently successful treatment of kala-azar or in rare cases, in the absence of clinical visceral leishmaniasis.
KA—Kala Azar (Visceral Leishmaniasis).
(PDF)
