# Supplementary figures and images for: Socioeconomic benefit to individuals of achieving 2020 targets for four neglected tropical diseases controlled/eliminated by innovative and intensified disease management: Human African trypanosomiasis, leprosy, visceral leishmaniasis, Chagas disease
Source: PLoS Negl Trop Dis. 2018 Mar 13;12(3):e0006250. doi: 10.1371/journal.pntd.0006250 (PMC5849290; doi:10.1371/journal.pntd.0006250)

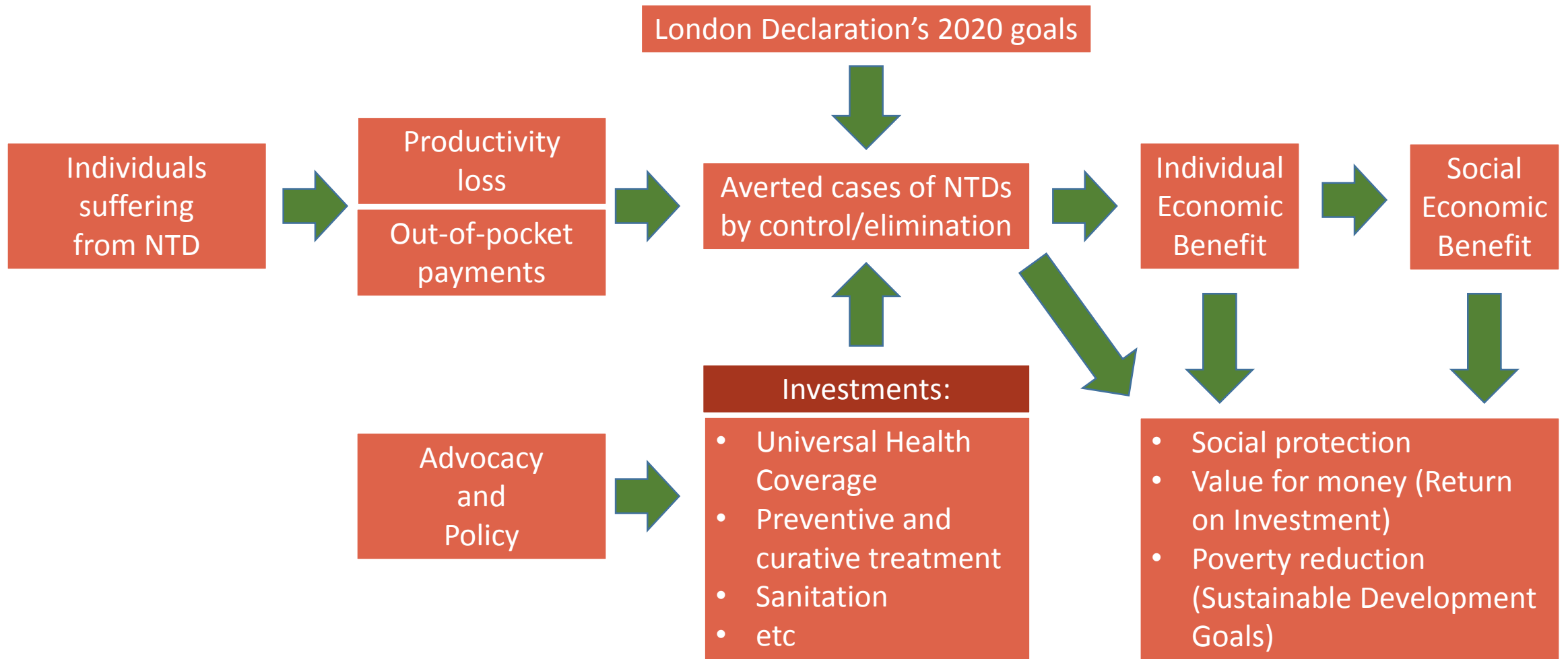

Supplement: S1 Fig — (PDF) [file pntd.0006250.s001.pdf]
