# Supplementary material for: Socioeconomic benefit to individuals of achieving 2020 targets for four neglected tropical diseases controlled/eliminated by innovative and intensified disease management: Human African trypanosomiasis, leprosy, visceral leishmaniasis, Chagas disease
Source: PLoS Negl Trop Dis. 2018 Mar 13;12(3):e0006250. doi: 10.1371/journal.pntd.0006250 (PMC5849290; doi:10.1371/journal.pntd.0006250)
Supplement: S1 File — (PDF) [file pntd.0006250.s002.pdf]

S2 Literature Search Syntax

Embase.com

('African trypanosomiasis'/de OR 'Chagas disease'/de OR 'trypanosomatid infection'/exp OR leprosy/exp OR 'Helminthiasis'/de OR 'Ascariasis'/de OR 'Trichuriasis'/de OR 'Hookworm infection'/exp OR 'Schistosomiasis'/exp OR 'Trachoma'/de OR 'Chlamydiasis'/de OR 'Chlamydiaceae infection'/de OR 'Dracunculiasis'/de OR 'Lymphatic filariasis'/exp OR 'Elephantiasis'/de OR 'Filariasis'/de OR 'Onchocerciasis'/de OR (((sleeping OR Hansen* OR neglected OR Robles) NEAR/3 (disease* OR sickness)) OR ((NTD* OR GWD) AND disease*) OR Chagas* OR leishmaniasis OR trypanosomiasis OR ((leishmania OR trypanosom* OR schizotrypanum OR worm* OR hookworm* OR whipworm* OR ancylostoma* OR Euglenoz* OR kinetoplast*) AND (infect* OR infestat* OR disease* OR transmiss*)) OR 'black fever' OR 'kala azar' OR lepros* OR lepra* OR helminth* OR ascari* OR trichuria* OR trichocephal* OR bunostomias* OR schistosom* OR bilharzi* OR 'Katayama fever' OR Trachoma* OR 'Egyptian ophthalmia' OR (chlamydia NEAR/3 conjunctiv*) OR dracuncul* OR draconti* OR filari* OR philar* OR wucher* OR brugia* OR elephantias* OR onchocerc* OR (river NEXT/1 blindness)):ab,ti) AND (productivity/de OR absenteeism/de OR 'job performance'/de OR 'return to work'/de OR 'work capacity'/de OR 'working time'/de OR 'cost of illness'/de OR 'patient transport'/de OR income/de OR salary/de OR 'medical leave'/de OR workload/de OR retirement/de OR employment/exp OR unemployment/de OR (((economic* OR financ* OR cost* OR pharmacoeconomic* OR expend* OR expens*) NEAR/3 (patient* OR individual* OR personal* OR household)) OR fee OR fees OR productivit* OR unproductivit* OR absenteeis* OR presenteeis* OR ((job OR work* OR profession* OR occupation* OR labour) NEAR/3 (perform* OR efficien* OR return* OR back OR capacit* OR abilit* OR disabilit* OR unab* OR limit* OR impair* OR loss OR losing OR restrict* OR reduct* OR input*)) OR (work* NEXT/1 (time OR week* OR day* OR load*)) OR workweek* OR workday* OR ((caregiver* OR illness* OR disease*) NEAR/3 burden) OR (distan* NEAR/3 (hospital* OR facilit* OR doctor* OR physician* OR 'health care')) OR 'patient transport' OR 'health shock' OR income* OR salary OR salaries OR payment* OR ((medical OR sick) NEXT/1 leave) OR workload* OR 'time off work' OR retire* OR employment* OR employed* OR unemploy* OR 'societal perspective' OR 'human capital' OR 'friction cost' OR 'lost time'):ab,ti) NOT ([animals]/lim NOT [humans]/lim)

Medline (OvidSP)

("Trypanosomiasis, African"/ OR exp "Chagas disease"/ OR exp "Euglenozoa Infections"/ OR exp leprosy/ OR "Helminthiasis"/ OR "Ascariasis"/ OR "Trichuriasis"/ OR exp "Hookworm infection"/ OR exp "Schistosomiasis"/ OR "Trachoma"/ OR "Chlamydiaceae Infections"/ OR "Chlamydia Infections"/ OR "Dracunculiasis"/ OR "Elephantiasis, Filarial"/ OR "Elephantiasis"/ OR "Filariasis"/ OR exp "Onchocerciasis"/ OR (((sleeping OR Hansen* OR neglected OR Robles) ADJ3 (disease* OR sickness)) OR ((NTD* OR GWD) AND disease*) OR Chagas* OR leishmaniasis OR trypanosomiasis OR ((leishmania OR trypanosom* OR schizotrypanum OR worm* OR hookworm* OR whipworm* OR ancylostoma* OR Euglenoz* OR kinetoplast*) AND (infect* OR infestat* OR disease* OR transmiss*)) OR "black fever" OR "kala azar" OR lepros* OR lepra* OR helminth* OR ascari* OR trichuria* OR trichocephal* OR

bunostomias* OR schistosom* OR bilharzi* OR "Katayama fever" OR Trachoma* OR "Egyptian ophthalmia" OR (chlamydia ADJ3 conjunctiv*) OR dracuncul* OR draconti* OR filari* OR philar* OR wucher* OR brugia* OR elephantias* OR onchocerc* OR (river ADJ blindness)).ab,ti.) AND ("Psychology, Industrial"/ OR absenteeism/ OR exp "Task Performance and Analysis"/ OR "underachievement"/ OR "return to work"/ OR "Work Capacity Evaluation"/ OR "cost of illness"/ OR exp "Transportation of Patients"/ OR exp income/ OR workload/ OR retirement/ OR employment/ OR unemployment/ OR "Health Services Accessibility"/ OR (((economic* OR financ* OR cost* OR pharmacoeconomic* OR expend* OR expens*) ADJ3 (patient* OR individual* OR personal* OR household)) OR fee OR fees OR productivit* OR unproductivit* OR absenteeis* OR presenteeis* OR ((job OR work* OR profession* OR occupation* OR labour) ADJ3 (perform* OR efficien* OR return* OR back OR capacit* OR abilit* OR disabilit* OR unab* OR limit* OR impair* OR loss OR losing OR restrict* OR reduct* OR input*)) OR (work* ADJ (time OR week* OR day* OR load*)) OR workweek* OR workday* OR ((caregiver* OR illness* OR disease*) ADJ3 burden) OR (distan* ADJ3 (hospital* OR facilit* OR doctor* OR physician* OR "health care")) OR "patient transport" OR "health shock" OR income* OR salary OR salaries OR payment* OR ((medical OR sick) ADJ leave) OR workload* OR "time off work" OR retire* OR employment* OR employed* OR unemploy* OR "societal perspective" OR "human capital" OR "friction cost" OR "lost time").ab,ti.) NOT (exp animals/ NOT humans/)

Cochrane

((((sleeping OR Hansen* OR neglected OR Robles) NEAR/3 (disease* OR sickness)) OR ((NTD* OR GWD) AND disease*) OR Chagas* OR leishmaniasis OR trypanosomiasis OR ((leishmania OR trypanosom* OR schizotrypanum OR worm* OR hookworm* OR whipworm* OR ancylostoma* OR Euglenoz* OR kinetoplast*) AND (infect* OR infestat* OR disease* OR transmiss*)) OR 'black fever' OR 'kala azar' OR lepros* OR lepra* OR helminth* OR ascari* OR trichuria* OR trichocephal* OR bunostomias* OR schistosom* OR bilharzi* OR 'Katayama fever' OR Trachoma* OR 'Egyptian ophthalmia' OR (chlamydia NEAR/3 conjunctiv*) OR dracuncul* OR draconti* OR filari* OR philar* OR wucher* OR brugia* OR elephantias* OR onchocerc* OR (river NEXT/1 blindness)):ab,ti) AND ((((economic* OR financ* OR cost* OR pharmacoeconomic* OR expend* OR expens*) NEAR/3 (patient* OR individual* OR personal* OR household)) OR fee OR fees OR productivit* OR unproductivit* OR absenteeis* OR presenteeis* OR ((job OR work* OR profession* OR occupation* OR labour) NEAR/3 (perform* OR efficien* OR return* OR back OR capacit* OR abilit* OR disabilit* OR unab* OR limit* OR impair* OR loss OR losing OR restrict* OR reduct* OR input*)) OR (work* NEXT/1 (time OR week* OR day* OR load*)) OR workweek* OR workday* OR ((caregiver* OR illness* OR disease*) NEAR/3 burden) OR (distan* NEAR/3 (hospital* OR facilit* OR doctor* OR physician* OR 'health care')) OR 'patient transport' OR 'health shock' OR income* OR salary OR salaries OR payment* OR ((medical OR sick) NEXT/1 leave) OR workload* OR 'time off work' OR retire* OR employment* OR employed* OR unemploy* OR 'societal perspective' OR 'human capital' OR 'friction cost' OR 'lost time'):ab,ti)

Web-of-science

TS=(((((sleeping OR Hansen* OR neglected OR Robles) NEAR/3 (disease* OR sickness)) OR ((NTD* OR GWD) AND disease*) OR Chagas* OR leishmaniasis OR trypanosomiasis OR ((leishmania OR trypanosom* OR schizotrypanum OR worm* OR hookworm* OR whipworm* OR ancylostoma* OR Euglenoz* OR kinetoplast*) AND (infect* OR infestat* OR disease* OR transmiss*)) OR "black fever" OR "kala azar" OR lepros* OR lepra* OR helminth* OR ascari* OR trichuria* OR trichocephal* OR

bunostomias* OR schistosom* OR bilharzi* OR "Katayama fever" OR Trachoma* OR "Egyptian ophthalmia" OR (chlamydia NEAR/3 conjunctiv*) OR dracuncul* OR draconti* OR filari* OR philar* OR wucher* OR brugia* OR elephantias* OR onchocerc* OR (river NEAR/1 blindness))) AND ((((economic* OR financ* OR cost* OR pharmacoeconomic* OR expend* OR expens*) NEAR/3 (patient* OR individual* OR personal* OR household)) OR fee OR fees OR productivit* OR unproductivit* OR absenteeis* OR presenteeis* OR ((job OR work* OR profession* OR occupation* OR labour) NEAR/3 (perform* OR efficien* OR return* OR back OR capacit* OR abilit* OR disabilit* OR unab* OR limit* OR impair* OR loss OR losing OR restrict* OR reduct* OR input*)) OR (work* NEAR/1 (time OR week* OR day* OR load*)) OR workweek* OR workday* OR ((caregiver* OR illness* OR disease*) NEAR/3 burden) OR (distan* NEAR/3 (hospital* OR facilit* OR doctor* OR physician* OR "health care")) OR "patient transport" OR "health shock" OR income* OR salary OR salaries OR payment* OR ((medical OR sick) NEAR/1 leave) OR workload* OR "time off work" OR retire* OR employment* OR employed* OR unemploy* OR "societal perspective" OR "human capital" OR "friction cost" OR "lost time")) AND (human* OR patient*))

Scopus

TITLE-ABS-KEY(((((sleeping OR Hansen* OR neglected OR Robles) W/3 (disease* OR sickness)) OR ((NTD* OR GWD) AND disease*) OR Chagas* OR leishmaniasis OR trypanosomiasis OR ((leishmania OR trypanosom* OR schizotrypanum OR worm* OR hookworm* OR whipworm* OR ancylostoma* OR Euglenoz* OR kinetoplast*) AND (infect* OR infestat* OR disease* OR transmiss*)) OR "black fever" OR "kala azar" OR lepros* OR lepra* OR helminth* OR ascari* OR trichuria* OR trichocephal* OR bunostomias* OR schistosom* OR bilharzi* OR "Katayama fever" OR Trachoma* OR "Egyptian ophthalmia" OR (chlamydia W/3 conjunctiv*) OR dracuncul* OR draconti* OR filari* OR philar* OR wucher* OR brugia* OR elephantias* OR onchocerc* OR (river W/1 blindness))) AND ((((economic* OR financ* OR cost* OR pharmacoeconomic* OR expend* OR expens*) W/3 (patient* OR individual* OR personal* OR household)) OR fee OR fees OR productivit* OR unproductivit* OR absenteeis* OR presenteeis* OR ((job OR work* OR profession* OR occupation* OR labour) W/3 (perform* OR efficien* OR return* OR back OR capacit* OR abilit* OR disabilit* OR unab* OR limit* OR impair* OR loss OR losing OR restrict* OR reduct* OR input*)) OR (work* W/1 (time OR week* OR day* OR load*)) OR workweek* OR workday* OR ((caregiver* OR illness* OR disease*) W/3 burden) OR (distan* W/3 (hospital* OR facilit* OR doctor* OR physician* OR "health care")) OR "patient transport" OR "health shock" OR income* OR salary OR salaries OR payment* OR ((medical OR sick) W/1 leave) OR workload* OR "time off work" OR retire* OR employment* OR employed* OR unemploy* OR "societal perspective" OR "human capital" OR "friction cost" OR "lost time")) AND (human* OR patient*))

CINAHL

(MH "Trypanosomiasis" OR MH Leishmaniasis OR MH leprosy OR MH "Helminthiasis+" OR MH "Ascariasis" OR MH "Hookworm infections" OR MH "Schistosomiasis+" OR MH "Trachoma+" OR MH "Chlamydiaceae Infections+" OR MH "Chlamydia Infections+" OR MH "Dracunculiasis" OR MH "Elephantiasis, Filarial+" OR MH "Elephantiasis" OR MH "Filariasis" OR MH "Onchocerciasis+" OR (((sleeping OR Hansen* OR neglected OR Robles) N3 (disease* OR sickness)) OR ((NTD* OR GWD) AND disease*) OR Chagas* OR leishmaniasis OR trypanosomiasis OR ((leishmania OR trypanosom* OR schizotrypanum OR worm* OR hookworm* OR whipworm* OR ancylostoma* OR Euglenoz* OR kinetoplast*) AND (infect* OR infestat* OR disease* OR transmiss*)) OR "black fever" OR "kala azar"

OR lepros* OR lepra* OR helminth* OR ascari* OR trichuria* OR trichocephal* OR bunostomias* OR schistosom* OR bilharzi* OR "Katayama fever" OR Trachoma* OR "Egyptian ophthalmia" OR (chlamydia N3 conjunctiv*) OR dracuncul* OR draconti* OR filari* OR philar* OR wucher* OR brugia* OR elephantias* OR onchocerc* OR (river N1 blindness))) AND (MH "Psychology, Occupational+" OR MH absenteeism+ OR MH "Task Performance and Analysis+" OR MH "Job Re-Entry+" OR MH "Job Performance" OR MH "Work Capacity Evaluation+" OR MH "Economic Aspects of Illness+" OR MH "Transportation of Patients+" OR MH income+ OR MH workload+ OR MH retirement+ OR MH employment+ OR MH unemployment+ OR MH "Health Services Accessibility+" OR (((economic* OR financ* OR cost* OR pharmacoeconomic* OR expend* OR expens*) N3 (patient* OR individual* OR personal* OR household)) OR fee OR fees OR productivit* OR unproductivit* OR absenteeis* OR presenteeis* OR ((job OR work* OR profession* OR occupation* OR labour) N3 (perform* OR efficien* OR return* OR back OR capacit* OR abilit* OR disabilit* OR unab* OR limit* OR impair* OR loss OR losing OR restrict* OR reduct* OR input*)) OR (work* N1 (time OR week* OR day* OR load*)) OR workweek* OR workday* OR ((caregiver* OR illness* OR disease*) N3 burden) OR (distan* N3 (hospital* OR facilit* OR doctor* OR physician* OR "health care")) OR "patient transport" OR "health shock" OR income* OR salary OR salaries OR payment* OR ((medical OR sick) N1 leave) OR workload* OR "time off work" OR retire* OR employment* OR employed* OR unemploy* OR "societal perspective" OR "human capital" OR "friction cost" OR "lost time")) NOT (MH animals+ NOT humans+)

PubMed publisher

((((sleeping[tiab] OR Hansen*[tiab] OR neglected[tiab] OR Robles[tiab]) AND (disease*[tiab] OR sickness[tiab])) OR ((NTD*[tiab] OR GWD[tiab]) AND disease*[tiab]) OR Chagas*[tiab] OR leishmaniasis[tiab] OR trypanosomiasis[tiab] OR ((leishmania[tiab] OR trypanosom*[tiab] OR schizotrypanum[tiab] OR worm*[tiab] OR hookworm*[tiab] OR whipworm*[tiab] OR ancylostoma*[tiab] OR Euglenoz*[tiab] OR kinetoplast*[tiab]) AND (infect*[tiab] OR infestat*[tiab] OR disease*[tiab] OR transmiss*[tiab])) OR black fever[tiab] OR kala azar[tiab] OR lepros*[tiab] OR lepra*[tiab] OR helminth*[tiab] OR ascari*[tiab] OR trichuria*[tiab] OR trichocephal*[tiab] OR bunostomias*[tiab] OR schistosom*[tiab] OR bilharzi*[tiab] OR Katayama fever[tiab] OR Trachoma*[tiab] OR Egyptian ophthalmia[tiab] OR (chlamydia[tiab] AND conjunctiv*[tiab]) OR dracuncul*[tiab] OR draconti*[tiab] OR filari*[tiab] OR philar*[tiab] OR wucher*[tiab] OR brugia*[tiab] OR elephantias*[tiab] OR onchocerc*[tiab] OR (river blindness[tiab]))) AND ((((economic*[tiab] OR financ*[tiab] OR cost*[tiab] OR pharmacoeconomic*[tiab] OR expend*[tiab] OR expens*[tiab]) AND (patient*[tiab] OR individual*[tiab] OR personal*[tiab] OR household[tiab])) OR fee[tiab] OR fees[tiab] OR productivit*[tiab] OR unproductivit*[tiab] OR absenteeis*[tiab] OR presenteeis*[tiab] OR ((job[tiab] OR work*[tiab] OR profession*[tiab] OR occupation*[tiab] OR labour[tiab]) AND (perform*[tiab] OR efficien*[tiab] OR return*[tiab] OR back[tiab] OR capacit*[tiab] OR abilit*[tiab] OR disabilit*[tiab] OR unab*[tiab] OR limit*[tiab] OR impair*[tiab] OR loss[tiab] OR losing[tiab] OR restrict*[tiab] OR reduct*[tiab] OR input*[tiab])) OR working time*[tiab] OR work week*[tiab] OR work day*[tiab] OR work load*[tiab] OR workweek*[tiab] OR workday*[tiab] OR ((caregiver*[tiab] OR illness*[tiab] OR disease*[tiab]) AND burden[tiab]) OR (distan*[tiab] AND (hospital*[tiab] OR facilit*[tiab] OR doctor*[tiab] OR physician*[tiab] OR health care[tiab])) OR patient transport[tiab] OR health shock[tiab] OR income*[tiab] OR salary[tiab] OR salaries[tiab] OR payment*[tiab] OR medical leave[tiab] OR sick leave[tiab] OR workload*[tiab] OR time off work[tiab] OR retire*[tiab] OR employment*[tiab] OR employed*[tiab] OR unemploy*[tiab] OR societal perspective[tiab] OR human capital[tiab] OR friction cost[tiab] OR lost time[tiab])) AND publisher[sb]

Google Scholar

(trypanosomiasis|Chagas|leprosy|Helminthiasis|Ascariasis|Trichuriasis|Hookworm|Schistosomiasis |Trachoma|Chlamydiasis|Dracunculiasis|filariasis|Onchocerciasis|"neglected disease") "(individual|personal|household) (cost|costs|economic|expenses|financial)"

Popline / Lilacs / Scielo

(trypanosomiasis OR Chagas OR leprosy OR Helminthiasis OR Ascariasis OR Trichuriasis OR Hookworm OR Schistosomiasis OR Trachoma OR Chlamydiasis OR Dracunculiasis OR filariasis OR Onchocerciasis OR "neglected disease") (productivity OR absenteeism OR "sick leave" OR unemployment OR "individual costs" OR "personal costs")

Google

(trypanosomiasis|Chagas|leprosy|Helminthiasis|Ascariasis|Trichuriasis|Hookworm|Schistosomiasis |Trachoma|Chlamydiasis|Dracunculiasis|filariasis|Onchocerciasis|"neglected disease") "(individual|personal|household) (cost|costs|economic|expenses|financial)" filetype:PDF

https://www.google.com.br/search?output=search&sclient=psy-ab&q=(trypanosomiasis%7CChagas%7Cleprosy%7CHelminthiasis%7CAscariasis%7CTrichuriasis%7CHookworm%7CSchistosomiasis%7CTrachoma%7CChlamydiasis%7CDracunculiasis%7Cfilariasis%7COnchocerciasis%7C%22neglected+disease%22)+%22(individual%7Cpersonal%7Chousehold)+(cost%7Ccosts%7Ceconomic%7Cexpenses%7Cfinancial)%22&oq=(trypanosomiasis%7CChagas%7Cleprosy%7CHelminthiasis%7CAscariasis%7CTrichuriasis%7CHookworm%7CSchistosomiasis%7CTrachoma%7CChlamydiasis%7CDracunculiasis%7Cfilariasis%7COnchocerciasis%7C%22neglected+disease%22)+%22(individual%7Cpersonal%7Chousehold)+(cost%7Ccosts%7Ceconomic%7Cexpenses%7Cfinancial)%22&gs_l=hp.3...27394.27394.0.27768.1.1.0.0.0.0.0.0..0.0.ernk_timecombined...1...1.1.32.psy-ab..1.0.0.lh0asBa9CXo&pbx=1&bav=on.2,or.r_qf.&bvm=bv.56988011,d.cWc,pv.xjs.s.en_US.dtklyhSMdi0.O&biw=1280&bih=671&dpr=1#newwindow=1&q=(trypanosomiasis%7CChagas%7Cleprosy%7CHelminthiasis%7CAscariasis%7CTrichuriasis%7CHookworm%7CSchistosomiasis%7CTrachoma%7CChlamydiasis%7CDracunculiasis%7Cfilariasis%7COnchocerciasis%7C%22neglected+disease%22)+%22(individual%7Cpersonal%7Chousehold)+(cost%7Ccosts%7Ceconomic%7Cexpenses%7Cfinancial)%22+filetype%3APDF
