# Supplementary material for: Socioeconomic benefit to individuals of achieving 2020 targets for four neglected tropical diseases controlled/eliminated by innovative and intensified disease management: Human African trypanosomiasis, leprosy, visceral leishmaniasis, Chagas disease
Source: PLoS Negl Trop Dis. 2018 Mar 13;12(3):e0006250. doi: 10.1371/journal.pntd.0006250 (PMC5849290; doi:10.1371/journal.pntd.0006250)
Supplement: S2 File — (PDF) [file pntd.0006250.s003.pdf]

S3 Grey Literature Search

The publications of the following organizations were searched: BMGF, Carter Center, CBM International (Christian Blind Mission), CDC Centers for Disease Control and Prevention, Centre for Neglected Tropical Diseases (Liverpool School of Tropical Medicine), Drug for Neglected Diseases Initiative, Global Alliance to Eliminate Lymphatic Filariasis, Global Network - NTD (Sabin Vaccine Institute), Hellen Keller International, Hollows, IDA Foundation, IDB - Interamerican Development Bank, IMA World Health, IMF, Imperial College London, International Trachoma Initiative, Lepra, Liverpool Associates in Tropical Health, PLOS NTD x economic, Research Triangle Institute – RTI, Sightsavers, The Task Force for Global Health, UN, USAID, WHO, World Bank, World Vision.
