# Supplementary material for: Socioeconomic benefit to individuals of achieving 2020 targets for four neglected tropical diseases controlled/eliminated by innovative and intensified disease management: Human African trypanosomiasis, leprosy, visceral leishmaniasis, Chagas disease
Source: PLoS Negl Trop Dis. 2018 Mar 13;12(3):e0006250. doi: 10.1371/journal.pntd.0006250 (PMC5849290; doi:10.1371/journal.pntd.0006250)
Supplement: S1 Table — The literature review yielded 11449 hits, 5316 after duplicates were excluded, for the 10 NTDs cited in the London Declaration. (PDF) [file pntd.0006250.s007.pdf]

S4 Results of database searches

Database	Hits	After exclusion of duplicates
**Embase.com**	2913	2854
**Medline (OvidSP)**	2887	682
**Web-of-science**	1224	478
**Scopus**	3339	660
**CINAHL**	282	126
**PubMed publisher**	175	150
**Cochrane**	60	7
**Popline**	176	147
**Lilacs**	257	100
**Scielo**	36	26
**Google Scholar**	100	87
**Total**	11449	5316
