# Supplementary material for: Socioeconomic benefit to individuals of achieving 2020 targets for four neglected tropical diseases controlled/eliminated by innovative and intensified disease management: Human African trypanosomiasis, leprosy, visceral leishmaniasis, Chagas disease
Source: PLoS Negl Trop Dis. 2018 Mar 13;12(3):e0006250. doi: 10.1371/journal.pntd.0006250 (PMC5849290; doi:10.1371/journal.pntd.0006250)
Supplement: S2 Table — The paucity of studies that provide quantitative estimates of productivity loss and OPPs from NTDs can be seen in the table above. (PDF) [file pntd.0006250.s008.pdf]

	Human African Trypanosomiasis	Chagas Disease	Visceral Leishmaniasis	Leprosy
**Papers screened on title and abstract**	95	355	497	709
**Assessed full text**	25	95	54	97
**Studies with quantitative data on productivity losses**	3	12	10	4
**Studies with quantitative data on out-of-pocket payments**	1	19	14	6
