# Supplementary material for: Socioeconomic benefit to individuals of achieving 2020 targets for four neglected tropical diseases controlled/eliminated by innovative and intensified disease management: Human African trypanosomiasis, leprosy, visceral leishmaniasis, Chagas disease
Source: PLoS Negl Trop Dis. 2018 Mar 13;12(3):e0006250. doi: 10.1371/journal.pntd.0006250 (PMC5849290; doi:10.1371/journal.pntd.0006250)
Supplement: S3 Table — NA—Not applicable NR—Not reported PAHO—Panamerican Health Organization. (PDF) [file pntd.0006250.s009.pdf]

S6 Publications reporting productivity loss for Chagas Disease

Author	Year	Country	Study design	Population	Sample size	Sequela	Definition of productivity loss	Results
Arata et al.	1994	Bolivia	report	NA	NA	b.chronic heart disease<br>c.heart failure<br>d.digestive disease	production lost due to chagas morbidity	b, c, d. conservative estimate of 25% incapacity is used for chronic Chagas cases in Bolivia<br>1-3% of chronic cases require surgery or sophisticated interventions
Basombrío et al.	1998	Argentina	Cost-benefit analysis	inhabitants of the province of Salta	NR	a.acute<br>b.chronic heart disease<br>c.heart failure<br>d.digestive disease	inability to work	a.average 7 days/year<br>b.mild cardiopathy cannot perform physically demanding jobs but can undertake light tasks, only partial incapacity - 14 days/year<br>c.patients with severe cardiopathy are incapable of working<br>d.full working ability for many years, partial incapacity only in advanced stages - 15 days/year<br>5% digestive cases need surgery
da Silva et al.	1995	Brazil	cross sectional	ambulatory patients	284	all	Percentage economically active per sex and phase of disease	Women<br>Phase I - 22%<br>Phase II - 27.6%<br>Phase III - 5.8%<br>Men<br>Phase I - 22%<br>Phase II - 15.2%<br>Phase III - 7.2%
Luquetti et al.	1997	Brazil	expert report	cases under evaluation for	NA	a.acute<br>b.chronic heart disease	inability to work	b. mild/moderate cardiopathy cases can work in any activity that does not require physical effort and assuming this does not present a

				disability benefit		c.heart failure<br>d.digestive disease		risk to themselves or others<br>c. severe cardiac cases are entitled to disability benefit and disability retirement<br>d. Digestive forms in principle do not contraindicate any work, only very severe cases or during recovery from eventual surgery
PAHO	2010	global	report	NA	NA	d. digestive disease	NR	d. 30% have normal bowel function
