# Supplementary material for: Socioeconomic benefit to individuals of achieving 2020 targets for four neglected tropical diseases controlled/eliminated by innovative and intensified disease management: Human African trypanosomiasis, leprosy, visceral leishmaniasis, Chagas disease
Source: PLoS Negl Trop Dis. 2018 Mar 13;12(3):e0006250. doi: 10.1371/journal.pntd.0006250 (PMC5849290; doi:10.1371/journal.pntd.0006250)
Supplement: S4 Table — CBA—cost-benefit analysis; CBA—cost-effectiveness analysis; n/a—not applicable; HH = household 1) 3 hospitals initially selected but 1 did not have any patients at time of treatment 2) PKDL Post–kala-azar dermal leishmaniasis, is a complication of visceral Leishmaniasis characterized by a macular, papular, or nodular rash, PKDL develops months to years after apparently successful treatment of kala-azar or in rare cases, in the absence of clinical visceral leishmaniasis. 3) definition of past and current patients: past PKDL case patient was defined as an individual with a macular, papular, or nodular rash for at least 1 month that was diagnosed by a physician and treated with SAG with resolution. A current PKDL case patient was defined as an individual with a macular, popular, or nodular rash for at least 1 month that was diagnosed by the study physician who was experienced in the clinical diagnosis of the disease. 4) excluded: pt kala-azar treatment failure or post-kala-azar-dermal leishmaniasis. (PDF) [file pntd.0006250.s010.pdf]

S7 Publications reporting productivity loss for Visceral Leishmaniasis

Author	Year	Country	Study design	Population	Sample size	Sequela	Definition of productivity loss	Productivity loss
Adhikari et al.	2005	Nepal (Danusha and Mahottari districts)	Observational	Economically active age group (15-59 years) and children below 15	18	VL	a. Days lost by patients & caregivers (incl. school days lost)<br>b. Days lost by patients (including school days lost)<br>c. Days lost by children and caregivers<br>d. Days lost by children<br>e. Opportunity costs	a. 178 days<br>b. 117 days<br>c. 67 days<br>d. 62 days<br>e. 8913.50 Rs
Adhikari et al.	2009	Nepal (Siraha and Saptari)	Observational	KA patients	61	VL	Total indirect costs of an episode of KA treatment	10910 (mean) 7800 (median)
Adhikari et al.	2010	Nepal	CBA	n/a	n/a	VL	a. Total opportunity cost of household (from Adhikari et al. 2009)<br>b. Productivity gains from KA interventions	a. 11000 Nepalese rupees<br>b. 26484 million Nepalese rupees (40% of total benefits)
Meheus et al.	2006	India (Bihar)	Observational	VL patients (including inpatients)	77	VL	a. Total indirect costs<br>b. Income loss (patient)<br>c. Income loss (attendant)<br>d. Monthly interest on loans<br>e. Days absent from school<br>f. Duration of illness	a. 5500 (median, rupees)<br>b. 4400 (median, rupees)<br>c. 900 (median, rupees)<br>d. 200<br>e. 67 days (25% were students)<br>f. 70 days (median)

Meheus et al.	2010	Indian subcontinent	CEA	n/a	n/a	VL	a. Productivity loss during VL treatment<br>b. Income loss during VL treatment	a. Assumed 100% productivity loss<br>b. Assumed an income loss of 1.48 US$/day.
Meheus et al.	2013	Sudan	Observational	Patients from two hospitals	75 (incl 45 under 15)	VL	a. Working days lost during a VL episode (patients)<br>b. Working days lost during a VL episode (caregivers)<br>c. Income loss, working individuals (n=22)<br>d. Income loss, all patients (n=75)<br>e. Income loss, all caregivers (n=99)	a. 51 (median)<br>b. 39 (median)(from 20/99 caregivers)<br>c. 101 US$ (median)<br>d. 41 US$ (mean)<br>e. 44 US$ (mean)
Ozaki et al.	2011	Bangladesh	Observational	Past and current patients with PKDL[2,3]	134 (56 patients treated, 78 untreated)	PKDL	a. Missed work days per PKDL treatment course<br>b. Missed work days per PKDL treatment (patients, caregivers)	a. 43 (median)<br>b. 123 (median)
Sarnoff et al.	2010	India (Bihar)	Observational	Households (HH) with a member currently with VL	194 HHs, 227 persons	VL	a. Annual number of working days lost<br>b. Annual income loss<br>c. Restricted daily activities	a. 120 days (amongst the 36% who worked)<br>b. 146 US$ (median)<br>c. 30 days (average) (all patients)
Sharma et al.	2006	Bangladesh	Observational	VL patients	113	VL	Income loss during VL episode	40 US$ (median)
Shreshta et al.	2008	Nepal (Kathmandu)	Observational	VL patients	60	VL	Working days lost due to the disease	100 (average, min 14 max 210)

Sundar et al.	2010	India (Bihar)	Observational	Patients who received VL treatment	183 patients, 171 HHs	KA	a. Duration of illness (symptoms to cure)<br>b. Monthly work loss after illness<br>c. Monthly work loss during illness<br>d. Median time loss during illness (the 15 weeks)	a. 15 weeks (median)<br>b. 1.08 weeks (before 4.29 weeks/month after illness 3.21 weeks/month)<br>c. 2.14 weeks/month<br>d. 2.14
