# Supplementary material for: Socioeconomic benefit to individuals of achieving 2020 targets for four neglected tropical diseases controlled/eliminated by innovative and intensified disease management: Human African trypanosomiasis, leprosy, visceral leishmaniasis, Chagas disease
Source: PLoS Negl Trop Dis. 2018 Mar 13;12(3):e0006250. doi: 10.1371/journal.pntd.0006250 (PMC5849290; doi:10.1371/journal.pntd.0006250)
Supplement: S5 Table — N.A.–Not applicable N.R.–Not reported. (PDF) [file pntd.0006250.s011.pdf]

S8 Publications reporting Out-of-Pocket Payments for Chagas disease

Author	Year	Country	Study design	Population	Sample size	Sequela	Definition of costs	OPPs
Abuhab et al.	2013	Brazil	prospective trial	adult patients high complexity cardiology university hospital in the city of São Paulo	58 Chagas and 519 other etiologies	Severe heart failure	costs of units spent during admission by each patient, including all fixed and variable costs of care at local costs, given the institutional division within which care was given. That methodology was used for accounting supplies (pharmacy, blood bank, and disposablematerial), procedures and surgeries, and diagnostic tests (imaging and blood)	mean cost per day US$ 467 (323–815)
Arata et al.	1994	Bolivia	report	NA	NA	a. acute<br>b. chronic heart disease<br>c. heart failure<br>d. digestive disease	annual costs of medical attention during the acute and the chronic phases and for some aspects of congenital Chagas	a. average cost about 100 Bolivianos<br>b. overall average: 200 Bolivianos<br>c.Severe cardiac cases: up to US$ 4000<br>d. 300 to 500 Bolivianos
Basombrío et al.	1998	Argentina	Cost-benefit analysis	population of the department of Anta, province of Salta	39 213	a. acute<br>b. chronic heart disease<br>c. heart failure<br>d. digestive disease	annual costs based on market prices and official standard fees for examination and basic treatment of patients.	a. US$ 591.8 b and c. US$ 603.62 d. US$ 736.15
Castillo-Riquelme et al.	2008	Colombia	Cost of illness, with Delphi consensus estimates	Chagas patients from	63	b. chronic heart disease<br>c. heart failure<br>d. digestive disease	Services consumed by the patients grouped into ambulatory care, visits to emergency services, in-patient bed-days, clinical and other diagnostic investigations or procedures, surgical procedures, and other medical procedures (e.g. rehabilitation if applicable). Basic, intermediate and high level of care.	b. basic US$46.4, intermediate US$ 188, high level care US$ 3651.5<br>c. basic US$ 51.4, intermediate US$ 259.3, high level care US$ 7980.9

Castillo-Riquelme et al.	2013	Chile	Cost-effectiveness analysis (model)	discharges between 2005 and 2009 with ICD-10 of chronic Chagas disease	49	b. chronic heart disease<br>c. heart failure<br>d. digestive disease	public annual costs and prices reported by the study of verification costs 2009 Chilean pesos June 2009	b. $6730 (pesos chilenos)<br>c. $470670 (pesos chilenos)<br>d. $733230 (pesos chilenos)
Lee et al.	2013	Latin America	simulation model (Markov)	NA	1000 individuals	not specified	Annual health care cost with an individual with T. cruzi infection	US$ 2600 ($1966 - 3034)
Medici et al.	2001	Bolivia	report	children under 5 years old, pregnant women and births in the infected areas	NR	acute	annual treatment for the target population	US$ 31
Moncayo et al.	1999	Latin America	report	NA	NA	not specified	Direct costs due to ambulatory medical care per patient per year in the countries of the Southern Cone Initiative	Argentina US$ 406<br>Brazil US$1,250<br>Bolivia US$227<br>Uruguay US$ 877<br>Weighted average cost six countries US$ 559
PAHO	1997	Chile	report	NA	NA	a. chronic heart disease<br>b. heart failure	Annual costs of treatment chronic chagasic cardiomyopathy	consultation US$ 411.5 - 549.5<br>Hospitalization US$ 170 (average 20 days)<br>Drugs US$ 168 - 906<br>Laboratory tests US$ 48.5
PAHO and others	2010	Latin America	report	NA	NA	acute	Costs of Benzonidazol treatment	US$ 10

Ramsey et al.	2014	Mexico	Markov decision model	NA	One million patients were simulated	a. acute<br>b. chronic heart disease<br>c. heart failure<br>d. digestive disease	Direct medical costs for CD include those for hospitalization, outpatient consultations, laboratory tests, annual screening, clinical procedures, and medications.	consultation/hospital/drugs/lab tests<br>a. US$ 17/6.5/61.2/12.4<br>b. US$ 98.1/21.4/162.8/36+25.4<br>c. US$ 481.2/151.2/162.8/254.4+179.9<br>d. US$ 296.6/93.2/162.8/156.8+110.9
Remme et al.	2006	Brasil and Argentina	book	NA	NA	not specified	Annual costs of consultations, care, and supportive treatment for chronic chagasic patients	US$ 1000
Schofield et al.	1991	Argentina, Brazil, Boilivia, Chile, Paraguay, Peru, Uruguay	Cost-benefit analysis (model)	NA	NA	b. chronic heart disease<br>c. heart failure<br>d. digestive disease	Average cost of consultation, care and supportive treatment for chrinic Chagas disease patients	c/d. hospitalization US$ 30.00 per patient per day (average of 25 days/year)<br>c. US$ 2500 for pacemaker<br>d. US$ 1750 grade III megaesophagus or mega-colon corrective surgery<br>c. US$185.00/year antiarrythmic drug (amiodarone)
Wilson et al.	2005	Latin America	Cost-effectiveness analysis (model)	NA	NA	a. acute<br>b. chronic heart disease<br>c. heart failure<br>d. digestive disease	annual costs from diagnosis and supportive treatment according to the type and severity	a. US$ 486<br>b. US$ 250<br>c. US$ 350.42<br>d.US$ 250
