# Supplementary material for: Socioeconomic benefit to individuals of achieving 2020 targets for four neglected tropical diseases controlled/eliminated by innovative and intensified disease management: Human African trypanosomiasis, leprosy, visceral leishmaniasis, Chagas disease
Source: PLoS Negl Trop Dis. 2018 Mar 13;12(3):e0006250. doi: 10.1371/journal.pntd.0006250 (PMC5849290; doi:10.1371/journal.pntd.0006250)
Supplement: S6 Table — N.A.–Not applicable VL—Visceral Leishmaniasis PKDL—Post–kala-azar dermal leishmaniasis, a complication of visceral Leishmaniasis characterized by a macular, papular, or nodular rash, PKDL develops months to years after apparently successful treatment of kala-azar or in rare cases, in the absence of clinical visceral leishmaniasis. KA—Kala Azar (Visceral Leishmaniasis). (PDF) [file pntd.0006250.s012.pdf]

S9 Publications reporting Out-of-Pocket Payments for Visceral Leishmaniasis

Author	Year	Country	Study design	Population	Sample size	Sequela	Defenition of costs	Direct Costs
Meheus et al.	2013	Sudan	survey	patients 2 hospitals	75	VL	a. *Direct medical costs in health-seeking phase* of VL episode (consultation, drugs, laboratory investigations)<br>b. *Direct non medical costs in healthseeking phase of VL episode* (transportation, food and other)<br><br>a. *Direct medical costs of admission and treatment of VL episode*<br>b. *Direct non-medical of admission and treatment of VL episode;* 85% caused by food costs of pt caretakers in the hospital<br><br>Total median direct expenditure by hh (incl health seeking phase and treatment facility)	a. 24.2 US$ (median), 45,9 US$ (mean)<br>b. 2,6 US$ (median), 15 US$ (mean)<br><br>a. 14 US$ (median), 18,5 US$ (mean)<br>b. 126,5 US$ (median) and 133 US$ (mean)<br><br>185 US$
Griekspoor et al.	1999	Sudan	Cost-effectiveness analysis and case study	Patients with VL	3067	VL	a. Costs per patient for recommended treament (pentavalent antimonium salt)	a. 100 US$ (on average)
Mannocci et al.	2007	Italy	Observarional (accessed hospital discharge data and mean length of stay)	patients with Leishmaniasis and Visceral Leishmaniasis who visited a hospital	N.A.	Leishmaniasis	Total hospitalisation costs for Leishmaniasis (ordinary hospitalization 3343,54 and daily hospitalization 329,50 multiplied with the respective number of discharges and added ordinary hospital costs to day-hospital ones)	a. 7,497,579.20 Euros (1,561,218.50 + 1,637,256.40 + 1,459,892.90 +1,468,983.60 + 1,370,227.80 for years 1999-2003)

Ozaki et al.	2011	Bangladesh	Observational (structured questionaires)	Past and current patients with PKDL	134 (55 patient treated, 78 untreated)	PKDL	a. total direct costs per treated patient because of treatement with SAG (inlc. diagnosis, provider fee, test, treatment, SAG, other medication, Syringe, informal payments to provider, transportation, miscellaneous, special food)<br>b. total direct costs per untreated patient (n=78)	a. 179 US$ (median) 218 (mean)<br>b. no direct expenditures
Sharma et al.	2006	Bangladesh	Observational (structured questionaires)	Patients with VL in two villages in Mymensingh District (the most highly endemic district in Bangladesh)	113 VL patients	VL	Total direct costs for care for a VL patient (inl. Provider, SAG, informal payments miscellaneous costs)	87 US$ (median)
Ahluwalia et al.	2003	Bangladesh (Fulbaria Thana)	Observational (focus group discussions, qualitative)	Patients with VL		VL	Amount of money needed for diagnosis and treatment	85-500 $
Sarnoff et al.	2010	India (Bihar)	Observation (household survey)	Sample of households from 80 villagesn. Households were included if any of the household members was currently suffering from VL	194 households, 227 individuals	VL	a. Direct and indirect costs prior to VL diagnosis (incl. treatments, transport, lodging)<br>b. Direct and indirect costs of VL treatment per VL episode (incl. VL test, treatment, transport lodging)<br>c. Additional expenses<br>d. median total treatment expenses	a. 18 US$ (median)<br>b. 85 US$ (median)<br>c. 101 US$ (median)<br>d. 131 per patient and 148 per household

Meheus et al.	2010	Indian subcontinent (India, Nepal, Bangladesh)	Cost-effectiveness analysis	N.A.	N.A.	VL	Drug costs', 'other direct medical' (incl contraceptives, administration, laboratory, inpatient bad-days) & 'nonmedical & indirect costs' of alternative strategies for VL per patient: a. L-AmB+MF<br>b. L-AmB+PM<br>c. MF+PM<br>d. SSG+PM<br>e. MF<br>f. PM<br>g. AmB<br>h. L-AmB10<br>i. L-AmB20<br>J. SSG	a. 95.7; 14.8; 12.8 US$ (2008)<br>b. 87.1; 20.5; 25.3 US$ (2008)<br>c. 29.5; 19.5; 23.8 US$ (2008)<br>d. 45.1; 29.9; 43.6 US$ (2008)<br>e. 62.8; 22.0; 45.4 US$ (2008)<br>f. 14.9; 30.6; 51.1 US$ (2008)<br>g. 20.9; 131.6; 45.4 US$ (2008)<br>h. 140; 11; 2.5 US$ (2008)<br>i. 280; 24.7; 6.9 US$ (2008)<br>j. 57.8; 40.7; 73.4 US$ (2008)
Meheus et al.	2006	India (Bihar)	Observational (Structured questionnaires and data from medical records)		77 patients (27 follow up, 50 inpatients)	VL	Out of pocket expenditures over the length of hospitalisation<br>a. Total<br>b. Medical costs (consultation, accommodation, investigations, medicines, medical supplies)<br>c. Non-medical (transport, food)<br><br>Prior to admission<br>a. Total<br>B. Medical cost<br>c. Non-medical	a. 3920 rupees (median)<br>b. 2510 rupees (median)<br>c. 1410 rupees (median)<br><br>a. 1220<br>b. 1090<br>c. 120

Sundar et al.	2010	India (Bihar)	Observational, semi-strucutered questionaire	patients who received VL treatment between september 2005 and september 2006	183 patients, 171 housholds	KA	a. Direct cost (inl. Provider, medicines, diagnostic, hospitalization)<br>b. Indirect (inl. Travel/food)<br>c. Total costs per patient (direct + indirect)	a. 83 US$ (median)<br>b. 33 US$ (median)<br>c. 127 US$ (median)
Olliaro et al.	2009	India (Bihar)	Cost-effectiveness analysis			VL	Costs of care and drug costs available for different treatment strategies (table 2 and 3) but health care perspective	
Adhikari et al.	2010	Nepal	Cost benefit analysis (discounted net benefits and internal rate of return)	N.A.	N.A.	VL	a. Average total direct costs (but based on other Adhikari et al. 2009)<br>b. Household savings of KA interventions	a. 7076 Nepalese Rupees<br>b. 14927 Nepalese Rupees (23% of total benefits)
Shretstah et al.	only abstract for poster presentation	Nepal (Kathmandu)	Observational	VL patients	60	VL	Working days lost due to the disease	100 (average, min 14 max 210)
Adhikari et al.	2009	Nepal (Siraha and Saptari)	Observational (questionaires)	KA patients	61	VL	Total direct costs of an episode of KA treatment (including medical costs 66,5%, food (22,6%), travel (8,9%) and other costs (1,9%)	7076 Napelese Rupees (average) 4805 (median)
Sharma et al.	2004	Nepal (Saptari and Siraha)	Observational (structured questionaires and focus group discussions)	KA patients	61 housholds	VL	Direct costs of treatment for one episode of KA, from the inception of the symptom to recovery (inl. Medical, transportation, food and other costs)	7076,23 Napalese Rupees (average)
Adhikari et al.	2005	Nepal (Danusha and Mahottari districts)	Observational (structured questionaires)	VL patients	18 patients	VL	Direct costs of a KA episode (inl. Medical costs (57%), food expensen (28%), transportation costs (5,4%), other costs (9,5%))	6853 Napalese Rupees (average)
